# Human surrogate models of central sensitization: A critical review and practical guide

**DOI:** 10.1002/ejp.1768

**Published:** 2021-05-08

**Authors:** Charles Quesada, Anna Kostenko, Idy Ho, Caterina Leone, Zahra Nochi, Alexandre Stouffs, Matthias Wittayer, Ombretta Caspani, Nanna Brix Finnerup, André Mouraux, Gisèle Pickering, Irene Tracey, Andrea Truini, Rolf‐Detlef Treede, Luis Garcia‐Larrea

**Affiliations:** ^1^ NeuroPain lab Lyon Centre for Neuroscience Inserm U1028 Lyon France; ^2^ Pain Center Neurological Hospital (CETD) Hospices Civils de Lyon Lyon France; ^3^ Department of Neurophysiology Mannheim center for Translational Neurosciences Heidelberg University Heidelberg Germany; ^4^ Nuffield Department of Clinical Neurosciences University of Oxford Oxford UK; ^5^ Department of Human Neuroscience Sapienza University Rome Italy; ^6^ Danish Pain Research Center Dept of Clinical Medicine Aarhus University Aarhus Denmark; ^7^ Institute of Neuroscience (IoNS) Université Catholique de Louvain (UCLouvain) Ottignies‐Louvain‐la‐Neuve Belgium; ^8^ Université Clermont Auvergne Neurodol Clermont‐Ferrand France

## Abstract

**Background:**

As in other fields of medicine, development of new medications for management of neuropathic pain has been difficult since preclinical rodent models do not necessarily translate to the clinics. Aside from ongoing pain with burning or shock‐like qualities, neuropathic pain is often characterized by pain hypersensitivity (hyperalgesia and allodynia), most often towards mechanical stimuli, reflecting sensitization of neural transmission.

**Data treatment:**

We therefore performed a systematic literature review (PubMed‐Medline, Cochrane, WoS, ClinicalTrials) and semi‐quantitative meta‐analysis of human pain models that aim to induce central sensitization, and generate hyperalgesia surrounding a real or simulated injury.

**Results:**

From an initial set of 1569 reports, we identified and analysed 269 studies using more than a dozen human models of sensitization. Five of these models (intradermal or topical capsaicin, low‐ or high‐frequency electrical stimulation, thermode‐induced heat‐injury) were found to reliably induce secondary hyperalgesia to pinprick and have been implemented in multiple laboratories. The ability of these models to induce dynamic mechanical allodynia was however substantially lower. The proportion of subjects who developed hypersensitivity was rarely provided, giving rise to significant reporting bias. In four of these models pharmacological profiles allowed to verify similarity to some clinical conditions, and therefore may inform basic research for new drug development.

**Conclusions:**

While there is no single “optimal” model of central sensitization, the range of validated and easy‐to‐use procedures in humans should be able to inform preclinical researchers on helpful potential biomarkers, thereby narrowing the translation gap between basic and clinical data.

**Significance:**

Being able to mimic aspects of pathological pain directly in humans has a huge potential to understand pathophysiology and provide animal research with translatable biomarkers for drug development. One group of human surrogate models has proven to have excellent predictive validity: they respond to clinically active medications and do not respond to clinically inactive medications, including some that worked in animals but failed in the clinics. They should therefore inform basic research for new drug development.

## 1 INTRODUCTION

Human experimental pain models play an important role in understanding physiological and pathological aspects of pain. They allow inducing temporarily a painful state in healthy subjects and thus offer an interim stage between animal models and clinical trials. This intermediate stage is critical, as human and rodent nociceptive systems show significant differences at peripheral (Schmelz & Petersen, 2001), thalamic (Barbaresi et al., 1986) and cortical levels (Evrard et al., 2014; van Heukelum et al., 2020), so that the data are often not transferable across species. Despite large preclinical investments, the overall clinical success of drugs for chronic pain remains low. Early phases of drug development routinely generate promises that are not confirmed in subsequent trials (London & Kimmelman, 2020), one prominent explanation being lack of cross‐species translation (Denayer et al., 2014). Being able to mimic aspects of pathological pain directly in humans has a huge potential not only to understand its physiopathology, but also to feed‐back animal research with translatable biomarkers for drug development.

While acute pain models are easy to produce using electric or thermal stimulation (Di Stefano et al., 2012), such acute stimuli have proved of little value to model neuropathic symptoms or signs in healthy humans, or to predict the effect of analgesic procedures (Bradley et al., 2016; Chapman et al., 1965; Weyer‐Menkhoff & Lötsch, 2018). Chronic neuropathic pain is characterized by a continuous or recurrent background component (ongoing or paroxysmal pain), associated in a variable proportion of cases with over‐reactivity to external stimuli reflecting peripheral or central sensitization mechanisms (Jensen & Finnerup, 2014). Reproducing ongoing neuropathic pain in healthy humans is challenging, because the initiating clinical event is damage to neural tissue, and there is no current human surrogate model that is able to produce continuous neuropathic pain without inducing unacceptable harm (Petersen et al., 2014; Schmelz, 2009). An alternative approach uses models that induce facilitation of nociceptive processing, and trigger central hyperexcitable states of which secondary mechanical hyperalgesia and dynamic mechanical allodynia are the measurable effects. The term ‘hyperalgesia’ denotes increased pain to stimuli that are normally painful (hence transmitted peripherally by nociceptive afferents), while ‘allodynia’ refers to pain elicited by a stimulus that normally does not cause pain, hence activating non‐nociceptive fibres. These two abnormal percepts occur in 20%–50% of patients with neuropathic pain (Jensen & Finnerup, 2014; Maier et al., 2010), and up to 70% in particular conditions such as post‐herpetic neuralgia (Johnson et al., 2010). Experimentally induced focal secondary hyperalgesia (2HA) and dynamic mechanical allodynia (DMA) are considered surrogates of neuropathic pain hypersensitivity: they are perceptually similar and share pain descriptors with their neuropathic counterparts, suggesting a commonality of underlying mechanisms (Gottrup et al., 2003; Jensen & Finnerup, 2014; Koltzenburg et al., 1994; Samuelsson et al., 2011), and sensory profiles observed in neuropathic pain can be mimicked by human surrogate models (Baumgärtner et al., 2002; Vollert et al., 2018). 2HA/DMA are defined as hypersensitive areas in non‐injured skin surrounding a real or simulated cutaneous injury, and there is ample consensus that they reflect central sensitization mechanisms (Arendt‐Nielsen et al., 2018; Klede et al., 2003; Koltzenburg et al. 1994; LaMotte et al., 1991; Rossler, 2013; Samuelsson et al., 2011; Schmelz, 2009; Simone et al., 1991; Torebjörk et al., 1992; Treede et al., 1992).

Some aspects of human surrogate models have been reviewed recently (van Amerongen et al., 2016; Vollert et al., 2018). The aims of the present systematic literature review were (1) to verify their ability to induce clinically relevant phenotypes of 2HA/DMA consistent with neuropathic central sensitization; (2) to analyse their success rate, spatial and temporal amplification; (3) to compare practical characteristics, reliability across studies and feasibility of these models for use in clinical trials, and (4) to compare their sensitivity to drugs. Our general objective is therefore to provide pain scientists with an updated analysis of the different possibilities to induce clinically relevant phenomena of central sensitization in humans, according to their experimental needs.

## 2 MATERIALS AND METHODS

### 2.1 Systematic search, data extraction and analysis

The review is an outcome of IMI‐PainCare (BioPain) initiative. and was conducted according to PRISMA with the following limitations: quantitative meta‐analysis was only possible for success rate and spatial amplification, while temporal amplification admitted semi‐quantitative analysis (see below). The remaining parameters were analysed qualitatively; bias reduction was only analysed for drug‐related RCTs. We searched from PubMed‐Medline, Cochrane, Google Scholar, WoS, Clinicaltrials.gov, using the terms “[**hyperalgesia OR allodynia**] **AND** [**human**] **AND** [**model**]”, limited to human models by introducing restricting terms **“**[**NOT** [**rat OR mouse OR rodent OR murine OR nonhuman**]”. Complementary searches were performed later in the process to tag electrical models: “[(**high frequency**) **AND (electrical) AND** (**stimulation**) **AND** (**hyperalgesia OR allodynia**)]”, as well as wind‐up ([(Wind‐up OR Windup) AND (capsaicin OR HFS OR LFS OR electric* OR freeze OR menthol OR “heat injury” OR “central sensitization” OR “human model”)], always with the above restricting terms. Iteratively, manual searches were conducted for reports not detected by automatic modes but available from the reference lists in literature. Searches were limited to English language, since database inception to March 31, 2020.

Three investigators (C.Q, A.K and L.G‐L) selected for further analysis publications reporting secondary hyperalgesia and/or allodynia in human surrogate pain models, and excluded papers reporting exclusively animal data, or exclusively primary hyperalgesia (i.e. within the area of induction only), or duplicate work. Work‐in‐progress was discussed during IMI PainCare‐BioPain meetings, then consecutive drafts were circulated among all co‐authors until a consensus was obtained on selected references, items to prioritize, conclusions and iconography. From each study, we extracted and summarized data on the characteristics of the sensitizing stimulus, the mode of assessment, details on outcome measures, consistency of responses across subjects, and –in studies on drug effects– whether blinding, randomization and placebo‐control were respected. These data are summarized in Tables 1, 2, 3, 4, 5, 6, and Supporting Information tables A&B.

**TABLE 1:** Summary of studies on intradermal Capsaicin

First author and year	*N*	Capsa dose (ug)	Pain on application (VAS)	2HA area (cm^2^)	DMA area (cm^2^)	Delay of max effect (min)	Pin‐prick force (g)	Responders (%)	Spatial amplification index*	Pharma intervention
Lötsch (2020)	16	100	–	–	–	–	26	–	–	Pregabaline (+)
Larsen (2018)	21	100	–	19.00	8.00	30.00	–	–	76.00	Diclofenac (−) & Methadone (+)
Andersen, Elberling, et al., (2017)	28	100	7.80	45.00	5.40	2.00	0.08–52	100.00	180.00	–
Diener (2017)	28	15	8.50	209.00	–	–	26	–	836.00	Botox A (+)
Ragavendran (2016)	63	250	–	–	–	–	26	–	–	Clonidine (+) & pentocyfylline (+)
Arout (2016)	18	250	–	–	–	–	21	–	–	Ethanol (−)
Wallace (2016)	19	250	8.00	40.00	–	20.00	–	–	160.00	Pregabalin (+) & T‐Ca^2+^ blocker (−)
Silberberg (2015)	15	100	–	54.58	22.70	–	–	100	218.32	–
Van Den Broeke (2015)	19	40	7.50	–	–	2.00	1.6–52	–	–	–
Nilsson (2014)	20	100	–	75.00	57	20	26/60	100	300.00	–
Wong (2014)	18	250	–	117.00	36.70	–	15	78.00	468.00	Pregabalin (+)
Hutchinson (2013)	12	50	5.60	83.80	70.00	15.00	–	–	335.20	–
Iannetti (2013)	11	60/120	9.50	–	–	–	13	90.00	–	–
Vuilleumier (2013)	16	100	9.60	44.70	23.30	120.00	52	100.00	178.80	Clobazepam (−) & clonazepam (−)
Kalliomäki (2013)	44	0.3	7.80	–	–	–	–	–	–	Cannabinoid (−)
Sumracki (2012)	18	10	–	52.70	–	–	–	–	210.80	Pregabaline (−) Minocycline (−)
Aykanat (2012)	14	10	–	3.14	–	–	26	–	12.56	–
Michaux (2012)	10	40	10	–	–	–	–	–	–	Cortisol (+)
Samuelsson (2011)	9	60/120	–	–	–	15.00	–	88.00	–	–
Gustorff (2011)	16	20	–	48.30	–	–	–	–	193.20	Lidocaine (+)
Andresen (2011a)	22	100	–	50.00	39	–	–	70.00	200.00	Buprenorphine (−) & Fentanyl (−)
Lam (2011)	13	10	–	13.00	11	–	140	–	52.00	Lidocaine (−)
Gustaffson (2009)	16	100/30/10/1	–	4.52	–	–	26	–	18.08	–
Lee (2008)	15	50	–	–	–	–	64/128/256/512	80.00	–	–
Klein, Magerl, et al., (2008)	18	40	–	–	–	–	0.08–52	88.00	–	Neramexane (+)
Wang (2008)	20	100	–	38.00	–	–	21	100.00	152.00	Pregabalin (+), Morphine (+), Diphenydramine (+)
Kraft (2008)	18	–	–	80.00	–	–	150	–	320.00	Cannabinoid (−)
Wallace (2008)	13	100	7	20.00	–	–	–	100.00	80.00	Gabapentine (−)
Gazerani (2007)	–	–	–	–	–	–	–	–	–	–
Wallace (2007)	15	100	–	37.00	16	–	15.00	93.00	148.00	Cannabinoid (−)
Geber (2007)	10	50	8.30	82.2	20.00	30.00	26.00	100.00	328.80	–
Pöyhia (2006)	9	250	–	16.38	–	–	164.00	100.00	65.52	Ketamine (+)
Scanlon (2006)	19	10/100	–	44.00	–	–	–	–	176.00	–
Gazerani (2006)	32	100	–	7.03	–	–	60	100	28.12	Botox A (+)
Gazerani (2005)	28	100	6.30	6.00	9	–	–	–	24.00	–
Pud (2005)	14	50	–	14.50	–	–	60	–	58.00	–
Gottrup (2004)	41	100	–	44.00	23.00	–	–	100.00	176.00	Gabapentin (+)
Wallace (2004)	14	100	–	35.00	20.00	–	15	–	140.00	Lamotrigine (–)
Yucel (2004)	12	50	6.51	48.00	39.00	–	31	100.00	192.00	–
Hughes, Macleod, et al. (2002)	12	250	–	63.00	31.00	–	20	–	252.00	–
Wallace, Ridgeway et al. (2002)	11	100	7.80	60.00	25.00	–	–	100.00	240.00	Ketamine (+) & Alfentanyl (+)
Eisenach (2002)	25	100	9.00	90.00	65.00	10.00	22	–	360.00	Adenosine (+)
Wallace, Barger et al. (2002)	13	100	–	24.00	–	–	15	92.00	96.00	Desipramine (−)
Yucel (2001)	10	50	7.80	–	–	–	75.9	60.00	0.00	–
Huang (2000)	11	50	–	93.00	–	–	20	90.00	372.00	–
Eisenach (2000)	24	100	–	70.00	34.00	–	22	–	280.00	Clonidine (+)
Witting (2000)	17	10	–	19.00	9.00	–	75.86	–	76.00	–
Gottrup (2000)	12	100	–	22.00	26.00	15.00	–	100.00	88.00	Ketamine (+) & lidocaine (+)
Ando (2000)	12	100	8.20	41.00	9.00	–	15	100.00	164.00	Mexiletine (+)
Koppert (2000)	12	2	9.20	16.50	6.00	12.00	45	–	66.00	Lidocaine (+)
Wasner (1999)	10	100	–	–	68.00	–	–	100.00	–	–
Baron (1999)	22	100	4.40	88.00	68.00	30.00	25	–	352.00	–
Magerl (1998)	12	40	6.80	–	–	–	26	83.00	–	–
Liu (1998)	12	100	–	59	20.00	–	–	–	236.00	–
Wallace (1997)	15	10	7.00	17.00	10.00	–	–	100.00	68.00	Lidocaïne (−)
Eisenach (1997)	6	100	8.20	47.00	28.00	7.00	–	–	188.00	Alfentanyl (+) & Amitriptyline (−) & Midazolam (−)
Kinnman (1997)	10	300	–	129.00	–	–	20	–	516.00	Morphine (+)
Ali (1996)	16	50	–	96.20	–	–	15	100	384.80	–
Park (1995)	12	250	–	19.00	6.00	–	–	–	76.00	Ketamine (+) & Alfentanil (+)
Treede (1993)	1	60	–	–	–	–	20	–	–	–
Lamotte (1991)	40	100	–	55.00	37.00	–	22.5	–	220.00	–
Simone (1989)	20	0.01/0.1/1/10/100	–	0/4/9.5/34/63	–	8.00	–	–	252.00	–

Note

Some of the studies in this table also dealt with other models and are therefore presented in other tables too. Note the lack of quantitative information in a significant proportion of studies. Pinprick force is given in grams (1g = 9.8 mN). VAS (app)= pain during application on a 0–10 scale. Values of VAS, delay of effects and 2HA/DMA surface areas are given as within‐study averages. *Spatial amplification index refers to the ratio [2HA area/Application area]. Symbols in the last column indicate the efficacy of the drug [+/−] to significantly abate hypersensitivity versus control/placebo in a given study.

**TABLE 2:** Summary of studies on topical Capsaicin

First author and year	*N*	Mode of appli.	Kindling	[C] (%)	Dur. of application (mins)	Appli. Area (cm^2^)	VAS (app)	2HA area (cm^2^)	DMA area (cm^2^)	Delay of max effect (min)	Pin‐prick force (g)	Resp. (%)	Spatial Amplif. index*	Pharma intervention
Ditre (2018)	66	solution	No	10	30	2.25	4.2	45.07	–	–	300	–	20.03	Nicotine withdrawal
Enax‐ Krumova (2017)	30	–	No	0.6	15	12	–	66	–	–	–	100	5.5	–
Niu, (2017)	28	cream	No	10	–	–	–	–	–	–	20.9	–	–	Acupuncture
Maracle (2017)	20	cream	No	0.075	30	–	3.30	–	–	10	0.08–52	80	–	–
Wanigasekera (2016)	24	cream	No	1	90	16	1.8	–	–	–	52	100	–	Gabapentin (+) & Ibuprofen (−)
Doll (2016)	8	patch	No	8	60	36	–	–	–	–	–	–	–	–
Kalliomäki (2013)	44	cream	No	0.075	90	16.5	7.8	–	16.5	–	15	–	–	–
Zheng (2009)	40	solution	No	–	30	3.14	–	40	–	–	26	–	12.73	Lidocaine (+)
Rukwied (2003)	20	solution	No	1	15	0.5	3	20	10	30	5.1	–	40	Cannabinoid (+)
Liu (1998)	12	patch	No	1	–	4	–	46	15.81	10	–	–	11.5	–
Morris (1997)	12	solution	No	0.1	30	0.5	–	200	–	45	–	–	400	NSAID (−)
Andersen (1996)	17	cream	No	1	–	4.9	–	25.9	–	–	–	–	5.28	Ketamine (+)
Kilo (1995)	24	solution	No	1	–	2.25	–	–	33.8	–	–	–	–	Ibuprofen (−)
Kilo (1994)	24	solution	–	1	–	1.5	–	47	24	–	23	–	16	–
Koltzenburg (1992)	20	solution	No	1	30	4	–	71.9	38	–	23	–	17.9	–
Linde (2019)	20	cream	Heat	0.1	30	50	–	–	–	30	–	–	–	–
Smith (2019)	79	cream	Heat	0.1	30	9	–	15	–	–	15	–	1.67	–
Price (2018)	24	cream	Heat	0.075	20	100	–	–	–	50	–	90	–	–
Arendt‐Nielsen (2016)	36	cream	Heat	1	30	9	–	–	–	–	60	–	–	Celecoxib (+) & AntiTRPV1 (−)
Rempe (2014)	16	solution	Heat	0.6	30	9	1.3	83	–	–	17	–	8.55	–
Liljencrantz (2014)	40	cream	Heat	0.075	–	9	–	–	–	–	25	–	–	–
You (2014)	78	solution	Heat	0.6	30	2.25	–	70	–	–	26	89	31.11	–
Cavallone (2013)	15	cream	Heat	1	30	9	4	40	26	–	–	100	4.44	Gabapentine (+)
Andresen (2011)	40	cream	Heat	0.075	30	9	–	48	–	30	–	–	5.3	–
Campbell (2011)	32	cream	Heat	10	–	6.25	–	47	–	–	21.5	–	7.52	–
Eisenach (2010)	14	cream	Heat	0.075	30	1	–	65	–	–	10	–	65	Ketorolac (−)
Bishop (2009)	12	solution	Heat	1	30	10.24	–	22.7	21.3	–	–	–	2.22	–
Frymoyer (2007)	23	cream	Heat	0.075	30	22.8	–	–	–	–	140	95	4.38	Dextrometorphan(−) & Morphine (+)
Mathiesen (2006)	27	cream	Heat	1	–	12.5	–	120.7	–	–	26	–	9.65	Gabapentine (+) & CHF3381 (+)
Jensen (2006)	85	cream	Heat	0.075	30	22.8	–	137	107	–	26	–	5.52	–
Zambreanu (2005)	12	cream	Heat	0.075	45	9	–	–	–	–	170	100	–	–
Iannetti (2005)	12	cream	Heat	0.075	–	9	4	–	–	–	–	100	–	Gabapentine (+)
Duedahl (2005)	25	cream	Heat	0.075	30	12.5	–	85	–	45	–	–	6.8	Dextrometorphan (+)
Maihöfner (2004)	11	solution	Heat	2.5	30	1.53	3.7	–	79.7	–	21.5	–	–	–
Dirks (2003)	20	cream	Heat	0.075	30	12.5	–	–	–	–	26	100	–	–
Petersen (2003)	23	cream	Heat	0.075	30	22.8	8	–	66	–	23	–	–	Morphine (+) Remifentanil (+) Lamotrigine (−)
Hood (2003)	10	cream	Heat	0.075	30	4	–	64	42	–	21.5	–	16	Remifentanil (+)
Eisenach (2002)	30	cream	Heat	0.075	–	4	3.2	60	37	–	–	–	15	Adenosine (+)
Dirks, Petersen, et al., (2002)	25	cream	Heat	0.075	30	12.5	–	97	–	–	–	–	7.76	Gabapentine (+)
Dirks, Møiniche, et al., (2002)	12	cream	Heat	0.075	30	12.5	–	200	–	–	40	–	16	Remifentanil (+)
Harding (2001)	13	solution	Heat	1	–	0.64	–	98	47.9	–	8.4	–	153.12	–
Petersen (2001)	14	cream	Heat	0.075	30	22.8	3.7	142	100	–	21	100	6.23	Remifentanil (+)
Yucel (2001)	10	cream	Heat	1	–	4	4.05	–	–	–	75.9	–	–	–
Dirks (2001)	23	cream	Heat	0.075	30	12.5	–	94	60	–	21.5	–	7.52	Adenosine (−)
Mikkelsen (2001)	25	cream	Heat	0.075	30	12.5	2.2	104	77	–	21.5	–	8.32	Magnesium (−)
McCormarck (2000)	10	solution	Heat	0.075	–	12.5	–	75	–	–	–	–	–	NSAID (−)
Dirks (2000)	25	cream	Heat	0.075	30	12.5	–	111	92	–	21.5	–	8.88	Lidocaine (+)
Petersen (1999)	10	cream	Heat	0.075	30	22.8	1.5	176	118	–	21	–	5.26	–

Note

Studies with and without heat‐kindling are presented separately. Please note that some of the studies in this table also dealt with other models and are therefore presented in other tables too. Pinprick force is given in grams (1g = 9.8 mN). Values of VAS, delay of effects and 2HA/DMA surface areas are given as within‐study averages. *Spatial amplification index refers to the ratio [2HA area/Application area]. Symbols in the last column indicate the efficacy of the drug [+/−] to significantly abate hypersensitivity versus control/placebo in a given study.

**TABLE 3:** Summary of studies using heat‐injury (thermode‐based) models

First author and year	*N*	Temp (C°)	Duration of appli. (min)	Thermode area (cm^2^)	Pin‐prick force (g)	2HA area (cm^2^)	DMA area (cm^2^)	Responders (%)	Delay of max effect (min)	Spatial amplification index*	Pharma intervention
Hansen (2018)	121	45°	3–5 (BTS)	12.5	50	448	–	–	–	35.84	–
Hansen (2017)	121	45°	3–5 (BTS)	12.5	19	448	–	–	–	35.84	–
Schifftner (2017)	18	40 to 45° (+1°)	15’ step	4	6	28	7	–	–	7	Alfentanil (−)
Hansen (2016)	54	45°	3–5 (BTS)	12.5	19	310.1	–	–	–	24.81	–
Rasmussen (2015)	17	47°	7	12.5	90	55	–	–	45	4.4	Hyperbaric oxygen (−)
Andersen, Gögenur, et al., (2015)	29	47°	7	12.5	13	41	–	–	60	3.28	Melatonin (−)
Ringsted (2015)	24	47°	7	12.5	51	30	–	87	–	2.4	–
Petersen (2014)	27	45°	3–5 (BTS)	15.7	26	345	–	100	–	21.98	Gabapentine (+) & Glutamate‐antagonist (+)
Jürgens (2014)	18	48°	10x 6sec	9	26	80	–	–	60	8.89	Acetaminophen (+)
Ravn (2014)	28	47°	7	12.5	90	–	–	–	–	–	Morphine (−)
Ravn (2013)	28	47°	7	12.5	90	25	–	–	–	2	Buprenorphine (+)
Bishop (2009)	27	45°	5.5	10.24	10	16	–	–	–	1.56	–
Petersen (2008)	60	45°	3–5 (BTS)	22.8	26	–	–	–	–	–	Morphine (+)
Stubhaug (2007)	12	47°	5	12.5	5.6	35	–	100	65	2.8	Ketolorac (+) & Methyloprednisolone (+)
Frymoyer (2007)	23	45°	3–5 (BTS)	22.8	26	–	–	–	–	–	Morphine (+) & Dextrometorphan (−)
Schulte (2005)	16	46°	7	12.5	45	33.5	–	–	–	2.68	Morphine (+)
Staud (2005)	44	45°	–	–	–	–	–	–	–	–	Dextrometorphan (+)
Yucel (2004)	12	47°	7	12.5	31	39.4	20.9	–	–	3.15	–
Dirks (2003)	20	45°	3–5 (BTS)	12.5	21.5	85	–	100	–	6.8	–
Werner (2002)	22	47°	7	12.5	45.5	85	41	86	–	6.8	Dexamethasone (−)
Dirks, Petersen, et al., (2002)	25										Gabapentine (+)
Dirks, Møiniche, et al., (2002)	12	45°	3 (BTS)	12.5	40	19	–	100	–	1.52	Remifentanil (+)
Brennum (2001)	25	47°	7	12.5	117	125	–	–	190	10	Naloxone (−)
Yucel (2001)	10	47°	7	3.75	75.9	–	–	50	–	–	–
Werner (2001)	22	47°	7	12.5	2	25	–	–	60	2	Gabapentine (+) & Dexamethasone (−)
Warncke (2000)	12	47°	7	12.5	5.6	65.5	–	–	–	5.24	Morphine (+) & Ketamine (+)
Mikkelsen (2000)	25	47°	7	12.5	117	86	–	100	60	6.88	Ketamine (+)
Lillesø (2000)	18	47°	7	12.5	43	50	–	–	50	4	Morphine (−)
Sjölund (1999)	10	47°	7	12.5	46	30	–	–	60	2.4	Adenosine (+)
Mikkelsen (1999)	25	47°	7	12.5	117	115	–	100	60	9.2	Naloxone (+) & ketamine (+)
Hammer (1999)	20	47°	7	12.5	42	40	–	–	60	3.2	Riluzole (−)
Pedersen (1998)	12	47°	7	12.5	34	85	28	100	120	6.8	–
Pedersen et al. (1998)	15	47°	7	12.5	46	85	–	100	60	6.8	Ketamine (−)
Petersen (1997)	20	47°	7	12.5	117	67.7	63.7	–	120	5.42	Ibuprofen (−)
Warncke (1997)	12	47°	6	12.5	–	50.2	–	100	30	4.02	Morphine (−) & Ketamine (+)
Ilkjaer (1997)	19	47°	7	12.5	117	80	42	–	60	6.4	Ketamine (−)
Warncke (1996)	20	47°	5	12.5	–	62	–	–	140	4.96	Ibuprofen (−)
Ilkjaer (1996)	25	47°	7	12.5	117	109	42	–	60	8.72	Dextrometorphane (+)
Brennum (1994)	10	47°	7	12.5	–	98	61	100	–	7.84	Morphine (+)
Pedersen (1994)	12	49°	5	3.75	16	38	–	100	180	10.13	Clobetasol propionate (−)
Moiniche (1994)	12	49°	5	3.75	16	53	–	91	180	14.13	Ketolorac (−)
Moiniche (1993)	12	49°	4	3.75	16	68	–	100	180	18 13	Piroxicam (−)
Dahl (1993)	18	50°	7	3.75	–	58	–	94	40	15.47	Lidocaine (+)
Cervero (1993)	10	39 to 42°	30’ step	–	5	14.2	–	100	–	–	–
Raja (1984)	8	53°	0.5	0.38	–	20.1	–	–	30	52.89	–

Note

A number of papers also dealt with other models, and are therefore presented in other tables too. Pinprick force is given in grams (1g = 9.8 mN). VAS (app) = pain during application on a 0–10 scale. BTS, brief thermal stimulation. Values of VAS, delay of effects and 2HA/DMA surface areas are given as within‐study averages. *Spatial amplification index refers to the ratio [2HA area/Application area]. Symbols in the last column indicate the efficacy of the drug [+/−] to significantly abate hypersensitivity versus control/placebo in a given study.

**TABLE 4:** Models based on repetitive electrical stimuli

First author	*N*	Frequency (Hz)	Dur. of application	VAS	2HA area (cm^2^)	DMA area (cm^2^)	Delay of max effect (mins)	Pin‐prick force (g)	Responders (%)	Spatial amplification index	Pharma intervention
Mauermann (2017)	16	2	120 min	6	27	–	15	26	–	–	Fentanyl (+)
Wehrfritz (2016)	20	2	155 min	6	50	40	30	45	95	166.6666667	Remifentanyl (+)
Nickel (2016)	48	1	45/60 min	5.6	15.6	–	–	26	–	–	Propofol (+)
Reindl (2016)	19	0.5/20	35 min	–	3	–	–	26	–	10	Neurostim (−)
Boyle (2014)	30	5	–	3.12	58	40	30	26	–	–	Gabapentine (+)
Tröster (2012)	15	2	150 min	5.5	31.2	–	2	45	–	104	Fentanyl (+)
Chu (2012)	10	2	210 min	5	–	–	–	12	–	–	Remifentanyl (+)
Nickel (2011)	12	2	35 min	–	26.6	–	2	26	–	88.67	Neurostim (+)
Lenz (2011)	16	2	150 min	6	44.1	–	30	26	100	147	Ketorolac (+) & remifentanyl (+)
Wehrfritz (2010)	20	2	180 min	6	45	–	30	26	–	150	Physostigmine (+) & N_2_O/O_2_ (+)
Bandschapp (2010)	14	2	180 min	4.2	64	50	25	26	–	64	Propofol (+)
Seifert (2010)	10	1	–	–	13.4	–	–	26	–	44.67	–
Seifert (2009)	12	1	50 min	4.92	46.76	–	–	26	–	155.87	Lidocaine (+)
Ayesh (2007)	24	5	20min	–	–	–	–	84	–	–	–
Geber (2007)	10	1	35 min	3.32	89.6	–	30	26	100	298.67	–
Chizh (2007)	32	2	180 min	6	44.69	38.44	40	26	–	148.97	Pregabalin (+) & Anti substance P (–)
Schulte (2005)	16	5	315 min	5	49.5	–	–	45	100	49.5	Morphine (+)
Chizh (2004)	20	1	155 min	5.8	28.1	37.4	30	45	–	93.67	Adenosine (+)
Pahl (2003)	10	5	120 min	5.9	35	20	30	45	100	–	Alfentanyl (+)
Klede (2003)	12	1	30 min	5	–	–	–	–	–	–	Lidocaïne (−)
Koppert (2001)	12	5	120 min	–	43	27.5	40	45	100	–	Ketamine (+), Alfentanyl (+), Lidocaine (+)
Van Der Broeke (2019)	15	100/42/20/5	45s/2min /4min /17min	–	50/80/60/35	–	–	14	–	3.98/6.56/ 4.78/2.78	–
Lenoir (2018)	18	100	45s	–	–	–	–	14	–	–	–
Cayrol (2018)	16	100	45s	–	–	–	–	14	–	–	–
Biurrun‐Manresa (2018)	17	100	45s	–	–	–	–	12	88	–	–
Reitz (2016)	10	100	45s	7.7	–	–	–	–	–	–	–
Van Der Broeke (2016)	14	100	45s	–	–	–	–	14	–	–	–
Henrich (2015)	20	100	45s	3.72	39	–	–	12	–	8.67	–
Xia (2016)	15	10/100/200	45s	–	–	55	30	13/30	–	–	–
Van Der Broeke (2014)	15	100	45s	–	–	–	–	15	–	–	–
Van Der Broeke (2014)	17	100	45s	–	–	–	–	15	–	–	–
Pfau (2011)	55	100	45s	4	–	–	83	–	83	–	–
Klein, Stahn, et al., (2008)	24	100	45s	4.9	9.89	–	–	–	79	2.82	–
Klein (2007)	8	100	45s	6.7	–	–	–	–	–	–	Ketamine (−)
Klein (2006)	13	100	45s	3.6	–	–	50	–	100	–	–
Klein (2004)	24	1/100	45s	5	38	–	45	0.02–51	–	–	–

Note

Upper part of the table: models using low‐frequency stimulation (LFS, 0.5–5Hz); lower part of the table: models using high‐frequency stimulation (HFS, >5Hz, mostly 100 Hz). Pinprick force is given in grams (1g = 9.8 mN Values of VAS, delay of effects and 2HA/DMA surface areas are given as within‐study averages. *Spatial amplification index refers to the ratio [2HA area/Application area]. Symbols in the last column indicate the efficacy of the drug [+/−] to significantly abate hypersensitivity versus control/placebo in a given study.

**TABLE 5:** Summary of the principal features of models

Models	Number of studies	Number of subjects	Conditioning stimulus						Secondary hyperalgesia (pinprick)							Amplification index				DMA	
			Duration (min, studies)		Area (cm^2^, studies)		Pain (0–10. studies)		Duration (h, studies)		Area (cm^2^, 95% CI, studies)			Responder rate (%, studies)		Spatial amp mean, 95% CI, studies			Temporal ampl. (min,med,max)	Area (cm^2^, studies)	
Intradermal capsaicin	61	1,063	~ 0.1	–	~0.25	–	7.8	22	nr	–	51.1	[40–62]	47	93.3	28	196.2	[161–231]	49	+++	28.1	30
Topical capsaicin	47	1,228	33.7	34	12.7	44	3.7	15	nr	–	70.2	[56–85]	27	95.8	11	17.6	[7–28]	29	+/++	53.3	19
Burn injury by heat	43	940	6.4	32	11.4	41	3.3	18	3–72	3	81.6	[53–111]	38	94.9	18	9.0	[6–12]	36	+++	38.2	8
Sunburn by ultraviolet light	28	490	nr	–	32.2	27	0.0	4	>36	14	44.1	[26–62]	18	49.5	13	5.1	[3–7.5)	13	+++	11.2	5
Low frequency electrical stimulation	21	378	124.4	18	0.4	15	5.2	16	2.0	18	39.7	[30–49]	18	99.2	6	117.1	[77–157]	13	+	36.19	7
High frequency electrical stimulation	15	281	0.8	14	9.6	11	5.0	7	5.2	3	34.2	[17–51]	4	87.7	4	5.2	[1.7–8.7]	3	+++	55	1

Note

Data averaged across reports are summarized for models with >10 published studies and reporting data from >100 subjects. Confidence intervals (95%) are provided for areas of secondary hyperalgesia and spatial amplification. “Studies” refer to the number of reports on which data are based. Duration and area of injection of intradermal capsaicin are estimations, as papers do not report these items. Note that in low‐frequency electrical (LFS) models the 2HA duration is the same as that of stimulus application. Due to lack of sufficient data, temporal amplification was semi‐quantitatively stratified on three levels: hypersensitivity duration less than two‐times (+), 2–10 fold (++) or >10 fold the conditioning time (+++). nr=not reported. Note the important reporting bias.

**TABLE 6:** Effects of drugs on the four principal human models of secondary hyperalgesia (Capsaicin, heat‐injury, UVB irradiation, LFS electrical stimuli) representing 90% of published data

Models Drugs	Capsaicin (Intra‐d. & topic.)		Heat (thermal injury)		Ultraviolet‐B (UV‐B)		Electric (LFS)	
Gabapentinoids	+	Lötsch (2020) (id) rc‐, *n* = 16 Wallace (2016) (id) rcb, *n* = 19 Wong (2014) (id) rcb, *n* = 13 Wang (2008) (id) rcb, *n* = 20 Gottrup (2004) (id) rcb, *n* = 41 Wanigasekera (2016) (t) rc‐, *n* = 24 Mathiesen (2006) (ht) rcb, *n* = 27 Iannetti (2005) (t) rcb, *n* = 12 Dirks (2002a) (ht) rcb, *n* = 25	+	Petersen (2014) rcb, *n* = 27 Dirks (2002a) rcb, *n* = 12 Werner (2001) rcb, *n* = 22 *(p =.06)*	+		+	Boyle (2014) rcb, *n* = 30 Enggaard (2010) rcb, *n* = 18 Chizh (2007) rcb, *n* = 32 Segerdahl (2006) rcb, *n* = 16
	_	Cavallone (2013) (ht) rcb, *n* = 15 Sumracki (2012) (id) rcb*, *n* = 18 Wallace (2008) (id) rcb, *n* = 13	_		**_**	Gustorff, Anzenhofer et al. (2004) rcb, *n* = 16	**_**	
Opioids	+	Wang (2008) (id) rcb, *n* = 20 Wallace (2002b) (id) rcb, *n* = 11 Eisenach (1997) (id) rcb, *n* = 46 Kinnman (1997) (id) rcb, *n* = 10 Park (1995) (id) rcb, *n* = 12 Larsen (2018) (id) rcb, *n* = 21 Frymoyer (2007) (ht) ‐cb, *n* = 23 Petersen (2003) (ht) rcb,*n* = 18 Hood (2003) (ht) ‐‐‐, *n* = 10 Petersen (2001) (ht) rcb,*n* = 14	+	Petersen (2008) rcb, *n* = 60 Frymoyer (2007) rcb, *n* = 23 Schulte (2005) rcb, *n* = 16 Dirks (2002b) ‐cb, *n* = 12 Warncke (2000) rcb, *n* = 12 Brennum (1994) r‐‐, *n* = 10	+	Ortner (2012) rcb, *n* = 32 Gustorff, Anzenhofer et al. (2004) rcb, *n* = 16	+	Mauermann(2017) rcb, *n* = 16 Wehrfritz (2016) rcb, *n* = 20 Tröster (2012) rcb, *n* = 15 Chu (2012) rcb, *n* = 10 Lenz (2011) rcb, *n* = 16 Schulte (2005) rcb, *n* = 16 Pahl (2003) r‐b, *n* = 10 Koppert (2001) rcb, *n* = 12
	**_**	Andresen (2011a ) (id) rcb, *n* = 22 (transdrm)	**_**	Schifftner (2017) rcb, *n* = 18 Ravn (2013) rcb, *n* = 28 Ravn (2014) rcb, *n* = 28 Lilleso (2000) rcb, *n* = 18 Warncke (1997) rcb, *n* = 12	**_**	Andresen (2011b) rcb, *n* = 22	**_**	
Anti‐NMDA (Oral/topical)	+	Klein (2008) (id) rcb, *n* = 18 Pöyhiä (2006) (id) rcb, *n* = 9 Mathiesen (2006) (ht) rcb, *n* = 27	+	Staud (2005), rcb, *n* = 44 Mikkelsen (1999) rcb, *n* = 25 Ilkjaer (1997) rcb, *n* = 19	+		+	
	**_**	Frymoyer (2007) (ht) rcb, *n* = 23 Kauppila (1995) (t) rcb, *n* = 8	**_**	Frymoyer (2007) rcb, *n* = 23 Hughes, Rhodes, et al. (2002) rcb, *n* = 12 Mikkelsen (2000) rcb, *n* = 25 Pedersen (1998b) rcb, *n* = 15	**_**		**_**	
Anti‐NMDA (iv, mainly ketamine)	+	Wallace (2002) (id) rcb,*n* = 11 Gottrup (2000) (id) rcb, *n* = 12 Park (1995) (id) rcb, *n* = 12 Duedahl (2005) (ht) rcb,*n* = 25 Andersen (1996) (t) rcb,*n* = 17	+	Hughes, Rhodes, et al. (2002) rcb, *n* = 12 Warncke (2000) rcb, *n* = 12 Mikkelsen (1999) rcb, *n* = 25 Warncke (1997) rcb, *n* = 20 Ilkjaer (1996) rcb, *n* = 25	+		+	Koppert (2001) rcb, *n* = 12
	**_**		**_**		**_**		**_**	
Lidocaine	+	Gustorff (2011) (id) rcb,*n* = 16 Gottrup (2000) (id) rcb, *n* = 12 Koppert (2000) (id) rcb,*n* = 12 Dirks (2000) (ht) ‐cb, *n* = 25 Zheng (2009) (t) rc‐, *n* = 40	+	Dahl (1993) r‐‐, *n* = 18	+	Gustorff (2011) rcb, *n* = 16 (topical inside spot)	+	Seifert (2009) rcb, *n* = 12 Koppert (2001) rcb, *n* = 12
	**_**	Wallace (1997) (id) rcb,*n* = 15 Lam (2011) (id) rcb, *n* = 13	**_**		**_**	Rossler (2013) rcb, *n* = 12 (topical outside spot)	**_**	Klede (2003) ‐‐‐, *n* = 12
Other NAch‐blockers	+	Ando (2000) (id) rcb, *n* = 12	+		+		+	
	**_**	Wallace (2004) (id) rcb,*n* = 14 Petersen(2003) (ht) rcb, *n* = 23	**_**		**_**		**_**	
Cannabinoids	+	Rukwied (2003) (t) rc‐, *n* = 20	+		+		+	
	**_**	Kalliomäki (2013) (id,t) rcb, *n* = 44 Kraft (2008) (id) rcb, *n* = 18 Wallace (2007) (id) rcb,*n* = 15	**_**	Redmond (2008) rcb, *n* = 17	**_**	Kraft (2008) rcb, *n* = 18	**_**	
NSAID	+	A‐Nielsen (2016) (t) rcb, *n* = 36 McCormack (2000) (t) rcb, *n* = 6	+	Stubhaug (2007) rcb, *n* = 12	+	Maihöfner (2007) rcb, *n* = 14 Sycha (2005) rcb, *n* = 42 Eisenach (2010) rcb, *n* = 14	+	Lenz (2011) rcb, *n* = 16
	**_**	Larsen (2018) (id) rcb, *n* = 21 Eisenach (2010) (ht) rcb, *n* = 14 Wanigasekera (2016) (t) rc‐, *n* = 24 Morris (1997) (t) ‐cb, *n* = 12 Kilo (1995) (t) rcb, *n* = 24	**_**	Petersen (1997) rcb, *n* = 20 Moiniche (1994) rcb, *n* = 12 Moiniche (1993) rcb, *n* = 12 Warncke (1996) rcb, *n* = 20	**_**	Lorenzini (2011) rcb, *n* = 12	**_**	
Tricyclic	+		+		+		+	Enggaard (2001) rcb, *n* = 18
	**_**	Wallace (2002a) (id) rcb, *n* = 13 Eisenach (1997) (id) rc‐, *n* = 6	**_**		**_**		**_**	
TRPV1 antagonist	+		+		+		+	
	**_**	A‐Nielsen (2016) (t) rcb, *n* = 36	**_**		**_**		**_**	
Adenosine	+	Eisenach (2002) (t) rcb, *n* = 30	+	Sjölund (1999) rcb, *n* = 10	+		+	Chizh (2004) r‐b, *n* = 20
	**_**	Dirks (2001) (ht) rcb, *n* = 23	**_**		**_**		**_**	
Adrenergic α−2	+	Ragavendran (2016) (id) rcb, *n* = 63 Eisenach (2000) (id) b, *n* = 24	+		+		+	
	**_**		**_**		**_**		**_**	
Botulinum Tox. A	+	Gazerani (2006) (id) rcb, *n* = 32	+		+		+	
	**_**	Diener (2017) (id) ‐‐‐, *n* = 28	**_**		**_**	Sycha (2006) rcb, *n* = 6	**_**	
Steroids	+	Michaux (2012) (id) rcb, *n* = 10	+	Stubhaug (2007) rcb, *n* = 12	+		+	
	**_**		**_**	Werner (2002) rcb, *n* = 22 Pedersen (1994) rcb, *n* = 12	**_**		**_**	
Paracetamol	+		+	Jürgens (2014) ‐cb, *n* = 18	+	Ortner (2012) rcb, *n* = 16	+	
	**_**		**_**		**_**	Lorenzini (2011) rcb, *n* = 12	**_**	
Glutamate antogonist	+		+	Petersen (2014) rcb, *n* = 27	+		+	
	**_**		**_**	Hammer (1999) rcb, *n* = 20	**_**		**_**	
Propofol	+		+		+		+	Bandschapp (2010) rcb,*n* = 14
	**_**		**_**		**_**		**_**	Nickel (2016) rcb, *n* = 48
20Hz‐neurostim.	+		+		+		+	Nickel (2011) rc‐‐, *n* = 12
	**_**		**_**		**_**		**_**	Reindl (2015) rc‐‐, *n* = 19

Note

The acronym “rcb” in brackets stands for “randomized, controlled, blinded”, when one of these was lacking and is replaced by a hyphen (“‐“). For capsaicin models, “id”: intradermal; “t”: topical; “ht”: heat‐topical. *=tested in patients. We included drugs tested in at least two independent studies with *n* > 10 subjects each. Studies were classed as “positive” if the drug affected significantly at least one output readout of hyperalgesia (e.g. 2HA area). Capsaicin, heat‐injury and LFS models responded to drugs clinically accepted for neuropathic pain (gabapentinoids, TCAs, iv ketamine, iv lidocaine) in a significantly greater proportion than to drugs with non‐clinical effect in NP (NSAIDs, oral anti‐NMDA, mexiletine, minocycline, steroids, anti‐TRPV1, magnesium, anti‐substance P, melatonin) (82.5% versus. 30.3%, *p* <.01).

We focused on mechanical hypersensitivity to gentle brushing (DMA: dynamic mechanical allodynia) and to punctate stimuli (pinprick hyperalgesia) since they are prevalent in neuropathic pain (Jensen & Finnerup, 2014; Maier et al., 2010), have long been recognized as hallmark signs of central sensitization (Treede et al., 1992), and were tested in a vast majority of published studies. Other mechanical stimuli (blunt pressure, impact stimuli) were not included because they are not unanimously acknowledged to reflect central sensitization (Göttrup et al., 2000; Kilo et al., 1994). Although more controversial, we included occasional reports describing changes in temporal summation of pain (“wind‐up”), either as signs of central sensitization or as models of central hypersensitivity (Andersen et al., 1996; Enggaard et al., 2001; Hughes et al., 2002).

Except for the rare cases where neurophysiological readouts were available, the methods used to assess “success” or “failure” of models were based on subjective reports of a “change in perception”. While we had to accept assessment as face value from each report, inconsistencies across studies were detected and discussed. As a quantitative measure of sensory amplification, we calculated the ratio between the area of secondary hyperalgesia and the area where the sensitizing stimulus was applied (“spatial amplification index”). When outcomes were measured repeatedly, we chose for statistics the time point showing highest area. A symmetrical measure of “temporal amplification” (duration of hypersensitivity relative to duration of the conditioning stimulus) could only be estimated semi‐quantitatively due to lack of data. Temporal amplification was therefore stratified on 3 levels, depending on whether hypersensitivity duration was less than 2‐times, 2–10 fold or >10 fold the conditioning time.

To avoid ambiguities in definitions, unless stated otherwise the term “allodynia” will be restricted here to *dynamic mechanical allodynia* (DMA), tested using brush stroke, and the term “secondary hyperalgesia” (2HA) will refer to responses to pinprick, even when pinprick force was not painful.

Hypothesis‐testing was used to compare 2HA induction ratios for different concentrations, thermode surfaces, duration of irradiation, etc. Relations between the areas of secondary hyperalgesia and areas of sensitizing stimuli were tested by linear or polynomial correlation models. Chi‐2 and confidence interval analyses were used to test possible associations between type of drugs tested and anti‐hyperalgesic effects.

Methodological quality of studies dealing with drug effects was assessed using the 5‐point Oxford Quality Scale (Jadad et al., 1996). A minimum of 10 subjects and score of 2B was required for inclusion. Risk of bias (Higgins et al., 2011) including no allocation concealment, lack of blinding (performance or detection bias), lack of control condition and reporting bias, was checked, and unless explicitly stated all included studies on drug effects were randomized and placebo‐controlled (Table 6). Absence of blinding was allowed if considered unavoidable (e.g. because of drug effects) and it did not decrease the level of evidence. Given the extreme heterogeneity in test stimulus, timing of assessment, specific readouts, etc., the evaluation of drug efficacy could not be expressed as VAS changes with confidence intervals but only in binary form (significant success/failure vs. placebo). When essential data were missing, we contacted authors to request additional information, and if these data could not be obtained we excluded those studies from further analysis.

## 3 RESULTS

**FIGURE 1:**
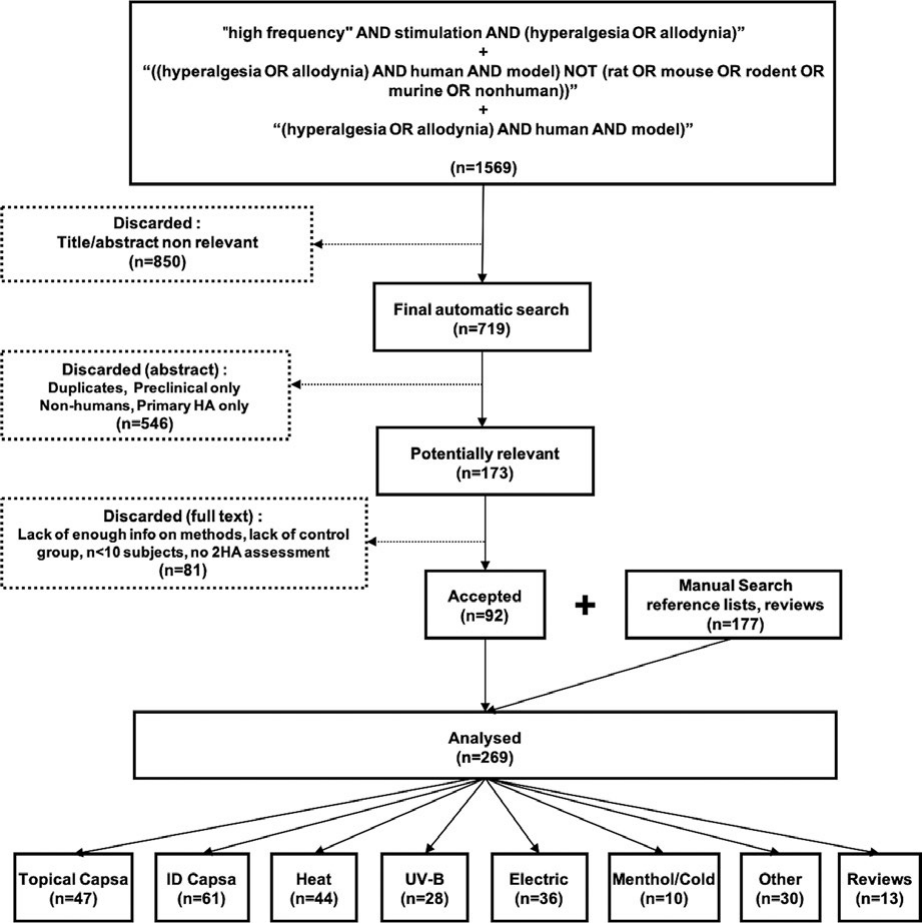
Flow Chart illustrating the paper selection procedure, and final number of articles retained according to specific models. Initial electronic search from databases (uppermost box) identified *n* = 1569 publications, of which 719 were potentially eligible from title/abstract. Exclusion of non‐human studies, studies limited to primary hyperalgesia and duplicates constrained the sample to *n* = 173 papers. A further 81 reports were excluded due to lack of enough information on methods/outcomes, lack of adequate control group or low sample size. The list was then completed by manual search with articles identified from reference lists and other sources, yielding a final dataset of *n* = 269 papers

Initial electronic search from databases identified *n* = 1569 publications, of which 719 were considered potentially eligible after a first analysis of title and abstract. Exclusion of duplicates, of studies limited to primary hyperalgesia and/or to animal models constrained the sample to *n* = 173 papers. Full text analysis of these (C.Q., L.G‐L.) led to exclusion of 81 reports due to (1) lack of enough information on methods or outcomes, (2) lack of adequate control in drug studies, or (3) anecdotal data or single case reports. This list was completed with articles identified from the reference lists in literature and other sources, yielding a final dataset of *n* = **269** papers. The selection flowchart and the number of papers by type of model are detailed in Figure 1.

We identified **108** studies reporting on the capsaicin models (61 intradermal, 47 topical); **72** on thermal or inflammatory injury models (44 heat, 28 UVB); **36** on electrically induced models (**21** low and **15** high‐frequency stimulation), and **40** reports on less prevalent models, including menthol/freeze (*n* = 10); nerve growth factor (NGF; *n* = 8), mustard oil or cinnamaldehyde (*n* = 6), hypertonic and acidic saline injections (*n* = 5), incisional models (*n* = 5), glutamate, endothelin‐1, lauryl sulphate or ciguatoxin (*n* = 6). In addition, **13** review papers on different aspects of models were also analysed.

### 3.1 Capsaicin‐based models

Capsaicin (8‐methyl‐N‐vanillyl‐6‐nonenamide) is an alkaloid found naturally in pepper (Nelson, 1919), which induces intense sensations of burn by its agonist effect on the transient receptor potential vanilloid‐1 (TRPV1) ion channel receptors (Bautista & Julius, 2008; Caterina et al., 1997; Gannon et al., 2016; Schmelz et al., 2000). The use of capsaicin as a surrogate model inducing secondary hyperalgesia is very common, with more than 100 original studies reported so far. Capsaicin delivery can be topical (Jancso, 1960) or via intra‐dermal injection (Simone et al., 1989); these modes of application lead to different patterns of effects at the application site and around it.

#### 3.1.1 Intradermal (ID) capsaicin

**FIGURE 2:**
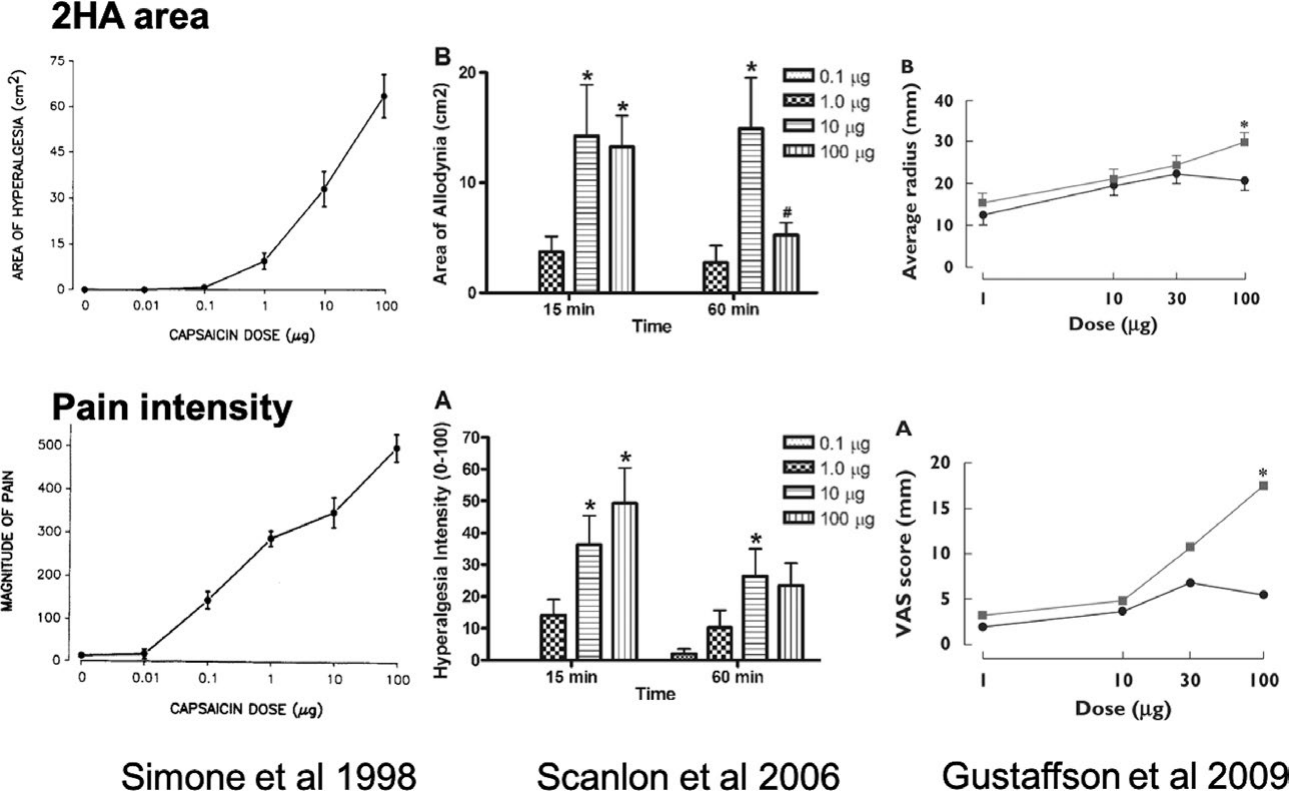
Dose–response graphs for intradermal capsaicin, as reported in three independent studies. Note the lack of significant effects of doses lower than 1 µg, and the inconsistent results of the 100 µg dosage, especially at 60 min

Secondary hyperalgesia surrounding an intradermal capsaicin injection is the only model where central sensitization has been conclusively shown to be the underlying mechanism in translational studies in humans and animals (Baumann et al., 1991; LaMotte et al., 1991; Simone et al., 1991; Torebjörk et al., 1992) (Table 1). A solution of capsaicin is injected intradermally, generally through a 30‐gauge needle, which is painful and technically more demanding than a simple subcutaneous injection. Previously suggested differences in 2HA area depending on injection site (Gazerani et al., 2007; Liu et al., 1998) could not be confirmed in this review. The quantity of injected capsaicin ranged from 0.01 µg to 300 µg, with preference for 50–100 µg (Table 1). A dose–response relationship was demonstrated repeatedly (Gustafsson et al., 2009; Scanlon et al., 2006; Simone et al., 1989), with 1 µg being the lowest dose producing a measurable hyperalgesic area. Due to capsaicin saturation properties, the gain of injecting more than 50 µg may be minimal, such doses yielding inconsistent results (Gustafsson et al., 2009; Figure 2). Responder rates (reported in 28/61 studies) were in most cases 75%–100% for 2HA, without significant difference between doses of 40–60, 100–120 or 250–300 µg of capsaicin (Table 1). Lower responder rates were reported for DMA at all the doses tested (Liu et al., 1998; Pöyhia & Vainio, 2006; Samuelsson et al., 2011). Geber et al. (2007) reported excellent test–retest reproducibility (*r* = 0.77) of stimulus‐response functions over two consecutive days.

The latency to develop 2HA/DMA after intradermal capsaicin is very short, from virtually no latency to a few minutes (Nilsson et al., 2014). Duration of effects increase with dose, from a few minutes for 1 µg to about 2 hr for 50–100 µg (Geber et al., 2007; Gottrup et al., 2004; Simone et al., 1989). No gender effects were reported in the limbs (Eisenach et al., 1997), but larger hyperalgesic areas in women relative to men, and in Indians relative to Caucasians were found in the forehead (Gazerani et al., 2005a,2005b, 2007).

**Gabapentinoids** decreased ID capsaicin hyperalgesic and/or allodynic area and pain intensity in five controlled studies on healthy subjects (Table 6). Subcutaneous or intravenous **opioids** also abated ID capsaicin hyperalgesia versus placebo in five studies (Table 6), while results were inconsistent for the transdermal route: positive for methadone/diclofenac (Larsen et al., 2018) but not for buprenorphine or fentanyl (Andresen, Staahl, et al., 2011). Intravenous **NMDA receptor antagonists,** including intravenous ketamine and intravenous ethanol decreased 2HA versus placebo (Arout et al., 2016; Gottrup et al., 2000; Park et al., 1995; Pöyhiä & Vainio, 2006), while oral naramexane decreased DMA but not pinprick hyperalgesia (Klein et al., 2008). **Lidocaine** inconsistently affected 2HA, both by intravenous route (decreased for Gottrup et al., 2000, Koppert et al., 2000; unchanged for Wallace et al., 1997) and by topical/regional routes (decreased for Zheng et al., 2009, Gustorff et al 2011a; unchanged for Koppert et al., 2000, Lam et al., 2011). Other sodium channel blockers such as lamotrigine, mexiletine and 4030W92 had little or no effect on 2HA (Ando et al., 2000; Wallace et al., 2004). Non‐replicated studies reported positive results with **hydrocortisone** (Michaux et al., 2012), intrathecal **adenosine** (Eisenach et al., 2002), and epidural or intrathecal **clonidine** (Eisenach et al., ,^1998^, ^2000^). Inconsistent results were observed with botulinum toxin (Diener et al., 2017; Gazerani et al., 2006). The **tricyclic antidepressants** amitriptyline and desipramine failed to modify 2HA in two studies (Eisenach et al., 1997; Wallace et al., 2002). No anti‐hyperalgesic effect was reported with oral **minocycline** (Sumracki et al., 2012), topical **ibuprofen** (Morris et al., 1997), **T‐type calcium channels blockers** (Wallace et al., 2016) or **cannabinoid** receptors (Kalliomäki et al., 2013; Kraft et al., 2008; Wallace et al., 2007). One study reported that unpleasantness, but not intensity, of capsaicin‐induced hyperalgesia was attenuated by THC (Lee et al., 2013).

No serious adverse effects have been reported so far. The main qualities of ID capsaicin are the short latency of 2HA and allodynia allowing rapid testing after injection, the sizeable duration and consistency of the effects, the minimal size of *primary* hyperalgesia and the high rate of responders. Such advantages may be offset by limitations such as the slightly invasive nature of the technique, the higher discomfort upon injection relative to its topical counterpart (e.g. Kraft et al., 2008), the difficulty to prepare lipophilic capsaicin in aqueous solution and target injections to the dermis layer (similar to tuberculine injection). Changes in blood pressure and heart rate have been documented, which may vary with injection at different depths in the skin (Silberberg et al., 2015).

#### 3.1.2 Topical capsaicin

We identified 47 studies using capsaicin applied topically in form of cream, solution‐soaked gauze or patch, generally during 30 min, on either the upper or the lower limb. Since the effects tend to be briefer than those of intradermal injection, most studies employed a heat sensitization procedure by applying a thermode at 40–45°C on the site of capsaicin application, which improves the stability of secondary hyperalgesia (Dirks & Petersen, 2003; Linde & Srbely, 2019; Petersen & Rowbotham, 1999) (Table 2). The duration of hyperalgesia was rarely indicated, but iterative application of cutaneous heat every 45 min proved useful to sustain the hypersensitivity during several hours in some studies (Dirks, et al., 2002; Modir & Wallace, 2010a; Petersen et al., 2001; Petersen & Rowbotham, 1999), although exceptions exists (e.g. Cavallone et al., 2013). Responder rate was 80%–100%, but information was provided in 11/47 studies only (Table 2).

A 2‐way factorial ANOVA with “mode of application” (solution vs. cream/patch) and “kindling” (yes/no) as factors, showed a significant enhancement of allodynic area for heat‐kindling (*F*(1,15) = 7.05; *p* =0.018) but no effect of application mode (*F*(1,15) = 0.19; *p* =0.6), and no interaction. Neither mode of application nor kindling influenced significantly the area of 2HA (Table 2).

**FIGURE 3:**
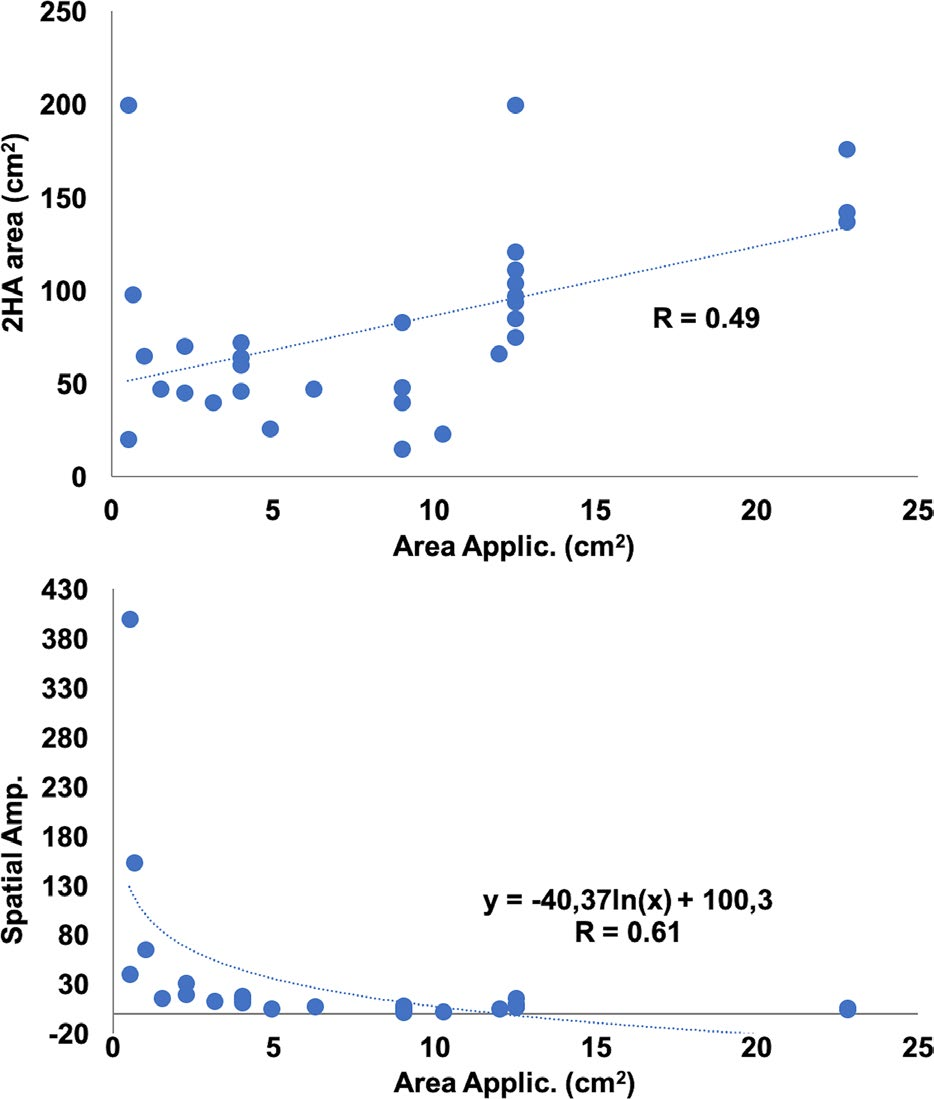
Relations between area of application and area of hyperalgesia in the topical capsaicin model. Data from the 31 studies providing enough data to compute correlations (see Table 2). Top: Positive correlation between the surface of topical application and the secondary hyperalgesic area. Bottom: Inverse exponential correlation between area of application and spatial amplification ratio of hyperalgesia: the ratio of surface amplification (2HA area / application area) decreases steeply with increasing surface of application

No correlation was found between the concentration of topical capsaicin and 2HA/DMA areas (*R* = 0.04; *p* =0.15; Table 2). Conversely, the application surface, which varied from 1 to 100 cm^2^, was positively correlated with the area of secondary hyperalgesia (Figure 3a), while the spatial amplification ratio (relation between 2HA area and surface of capsaicin application) declined exponentially with increased application surfaces (Figure 3b).

The hyperalgesic effects of topical capsaicin were attenuated by **gabapentin** 1200–1800 mg, versus placebo in 4 out of 5 studies (Cavallone et al., 2013; Dirks et al., 2002; Iannetti et al., 2005; Mathiesen et al., 2006; Wanigasekera et al., 2016). The **opioids** morphine, fentanyl and remifentanil decreased 2HA in one open and 3 placebo‐controlled studies (Frymoyer et al., 2007; Hood et al., 2003; Petersen et al., 2001, 2003). While opioids reduced both 2HA and physiological nociception, gabapentin did not affect nociceptive thresholds in normal skin. One study found the N‐methyl‐D‐aspartate (NMDA) receptor antagonist **ketamine** to decrease heat/capsaicin‐induced 2HA, nociceptive reflexes and wind‐up responses versus placebo (Andersen et al., 1996). Other NMDA antagonists such as **dextromethorphan** reduced 2HA when applied intravenously (0.5 mg/kg) (Duedahl et al., 2005) but not by oral route (30–100 mg; Kawamata, et al., 2002; Frymoyer et al., 2007). Intrathecal **adenosine** reduced 2HA *and* DMA areas (Eisenach et al., 2002), but was ineffective by systemic route (Dirks et al., 2001). Topical **cannabinoid** agonists gave inconsistent results, positive versus placebo for Rukwied et al., (2003) but not for Kalliomäki et al., (2013). Nonsteroidal anti‐inflammatory drugs (**NSAIDs**) were inactive on 2HA/DMA when administered systemically (Kilo et al., 1995; Wanigasekera et al., 2016) or intrathecally (Eisenach et al., 2010), but reduced DMA if administered topically (McCormack et al., 2000). Capsaicin‐induced 2HA was not attenuated beyond placebo by the sodium channel blocker **lamotrigine** (Petersen et al., 2003), the **TRPV1 antagonist** V116517 (Arendt‐Nielsen et al., 2016) or intravenous **magnesium** (Mikkelsen et al., 2001).

Topical capsaicin is a safe and easy‐to‐use model of hyperalgesia. No serious adverse effects have been reported. Main qualities are ease of handling and very moderate pain at induction relative to ID capsaicin. Limitations may be the unfavourable ratio between the duration of application and the duration of hyperalgesia, as well as the relatively low spatial amplification factor. These difficulties can be improved by heat‐kindling, which is the recommended procedure to enhance stability and duration of hypersensitivity.

### 3.2 Heat injury models

Initial techniques using small skin spots at >50°C on glabrous skin were instrumental in clarifying many aspects of peripheral encoding of heat pain (Meyer & Campbell, 1981; Raja et al., 1984) (Table 3), but produced second degree burns, blisters and visible oedema, and have been replaced by techniques using lower temperatures on hairy skin. Prolonged thermal stimulation at non‐painful levels (40–42°C) can also trigger secondary hyperalgesia; however, the stimulus has to be maintained for long periods, and hyperalgesia is extremely short‐lived (Cervero et al., 1993; Schifftner et al., 2017). Therefore, most studies used thermodes at higher temperatures (~47°C) applied during 5–7 min to a 9–16 cm^2^ hairy skin contact area (Table 3). This results in a first‐degree burn injury (redness without blistering) for less than one day, primary hyperalgesia on the site of exposure and 2HA/DMA in adjacent tissue. Areas of 2HA were quite variable (95% CI: 53–111), with average surface amplification index ~9 (95% CI 5.59–11.7). Maximum effect is reached at about 75 min [95% CI 54–93] and the duration of hyperalgesia (reported in very few studies) could range from 3 to 72 hr. Most studies reported high 2HA response rates (80%–100%), while the incidence of allodynia was only ~60% (Hammer et al., 1999; Werner et al., 2001). (all calculations from data in Table 3).

The “brief thermal stimuli” variant (Dirks et al., 2002, 2003), uses 1°C/sec temperature increase from 32°C to 45°C, and tests hyperalgesia after 3 min at 45°C. Although this procedure provides large areas of hypersensitivity and high responder rates (200–300 cm^2^, 100%), the duration of hyperalgesia is very short, and assessment has to be performed with the thermode still in place (Dirks et al., 2003; Hansen et al., 2017). Using a different approach (60 heat‐pulses at 48°C for 6 s) Jurgens et al., (2014) reported large areas of 2HA/DMA (80 cm^2^) with maximal effect at 60 min and 8‐hr duration, but the procedure has not been replicated.

Thermode‐induced 2HA was decreased by **systemic opioids** including morphine, buprenorphine and alfentanil in seven studies (six controlled; Table 6), but failed to overpower placebo in four controlled trials (Lillesø et al., 2000; Ravn et al., 2014; Schifftner et al., 2017; Warncke et al., 1997). **Gabapentin** decreased 2HA in two controlled studies (Dirks et al., 2002; Petersen et al., 2014) and showed a trend in a third one (*p* =.06; Werner et al., 2001). The NMDA receptor antagonist **ketamine** had consistent effects on 2HA when administered intravenously (Hughes, Rhodes, et al., 2002; Ilkjaer et al., 1996; Mikkelsen et al., 1999; Warncke et al., 1997, 2000), but not via subcutaneous or oral routes (Mikkelsen et al., 2000; Pedersen et al., 1998). Oral **dextromethorphan** did not modify brush‐evoked allodynia, barely decreased pinprick hyperalgesia (Ilkjaer et al., 1997), had inconsistent results on temporal summation (wind‐up) (Hugues, Rhodes, et al., 2002; Staud et al., 2005), and added to morphine did not modify results relative to morphine alone (Frymoyer et al., 2007). Non‐replicated reports described efficacy of **acetaminophen** (Jürgens et al., 2014), intravenous **adenosine** (Sjölund et al., 1999), glutamate receptor antagonist LY545694 (Petersen et al., 2014) and hyperbaric oxygen (Rasmussen et al., 2015). Pre‐emptive local infiltration of **lidocaine** postponed but not prevented 2HA (Dahl et al., 1993), and **steroids** yielded conflicting results, negative for topical clobetasol and iv dexamethasone (Pedersen et al., 1994; Werner et al., 2002) but positive for iv methylprednisolone (Stubhaug et al., 2007). **NSAIDs** did not reduce heat‐injury 2HA (Moiniche et al., 1993, 1994; Petersen et al., 1997; Warncke et al., 1996) except when administered intravenously (Stubhaug et al., 2007). Intravenous **melatonin** 10‐100mg and the cannabinoid analogue **nabilone** (tetrahydrocannabinol‐THC) were ineffective (Andersen, Poulsen, et al., 2015; Redmond et al., 2008).

Merits of classical thermode‐based procedures (47°C, 7 min) are the ease and speed of the technique, long duration of 2HA and relatively good consistency across studies. Disadvantages are the high cost of the thermode, the potential epidermal injuries if applied for long, and the inter‐subject variability of 2HA surface. The ‘brief induction’ techniques might be less prone to induce epidermal lesions. Late hyperpigmentation in the area may occur in 1%–2% of participants, and blistering in up to 25% when using thermodes 12.5 cm^2^ or larger (Dahl et al., 1993; Pedersen et al., 1998; Sjölund et al., 1999; Dirks et al., 2003), and even with smaller thermodes if temperature is pushed to 50°C (Dahl et al., 1993). Although very rare, severe thermal injury has been described following the use of malfunctioning, overheating contact thermodes (Springborg et al., 2016).

### 3.3 Ultraviolet‐induced inflammation techniques (Supporting Information Table A)

These models were developed in the mid‐1990s to induce inflammation‐related hyperalgesia in animals, then in humans (Benrath et al., 1995; Bickel et al., 1998). The technique is based on skin irradiation with a source of ultraviolet type B (UVB) at 290‐320nm. All protocols use the notion of “Minimal Erythema Dose” (MED), which is the minimal dose of irradiation to induce a visibly irritated red area (Hoffmann & Schmelz, 1999). MEDs are generally determined 1–7 days before the experiments, using five circular spots of 1.5cm with growing duration of irradiation from a calibrated UVB source (Modir & Wallace, 2010b). On the experimental day a single spot of 1.5–5 cm diameter is irradiated, in general at 3 MED, which provides better reliability than 1–2 MED (Bickel et al., 1998; Siebenga et al., 2019). Skin areas show no alterations immediately after UVB exposure, and neither spontaneous pain nor allodynia are described. An erythema develops at about 6 hr after irradiation and reaches maximum intensity after 12–36 hr. At this point *primary* hyperalgesia is a constant feature, while 2HA/DMA are inconsistent, with large variations in published reports (0–137 cm^2^, Supporting Information Table A). When they develop, 2HA/DMA attain their maximum 16–24 hr after irradiation and may last up to 4 days. Spatial amplification index in 13 papers providing quantitative data was 5.1 [95%CI 3–7.5]; however, at least seven studies failed to detect any significant 2HA outside the irradiated spot, and in two it was obtained only after heat rekindling (Eisenach et al., 2010; O’Neill et al., 2015) (Supporting Information Table A). Intra‐ and inter‐individual variation coefficients of 2HA area were reported to be 23% and 46%, respectively (Lorenzini et al., 2012). The rate of 2HA induction, when reported, ranged from 0% to 100% (Supporting Information Table A).

Drugs found effective to decrease UVB‐induced 2HA in controlled studies (Table 6) included **NSAIDs** (Eisenach et al., 2010; Maihöfner et al., 2007; Sycha et al., 2005), **paracetamol**/**tramadol** (Ortner et al., 2012) and systemic **opioids** (Gustorff, Hoechtl, et al., 2004). Topical **lidocaine** was effective if applied within the irradiated spot, but not when infused just outside it (Gustorff, et al., 2011; Rössler et al., 2013). Regional/transdermal **opioids** (Andresen, Staahl, et al., 2011), **paracetamol/ketorolac** (Lorenzini et al., 2011) and **botulinum** toxin (Sycha et al., 2006) failed to modify 2HA.

**Gabapentin** 600 mg (Gustorff, Hoechtl, et al., 2004) and Tetrahydrocannabinol/cannabidiol (THC/CBD) (Kraft et al., 2008), did not modify UVB‐related 2HA. One single study found a significant effect of **benzodiazepines** clobazam and clonazepam on UVB‐induced secondary hyperalgesia (Besson et al., 2015), but a nonspecific effect on vigilance could not be excluded.

The main qualities of UV‐B sensitization are the long duration of effects and the absence of ongoing pain. Limitations are the need to calibrate the MED at least 24 hr before sensitization, the long time needed for induction of symptoms, the possibility of hyperpigmentation in the irradiated spot in >50% of subjects, and even up to 3 years after exposure (Siebenga et al., 2019), and the inconsistent development of secondary hyperalgesia.

### 3.4 Models based on electrical stimulation

Nociceptive long‐term potentiation (LTP) leads to amplification of synaptic signals and is thought to be at the basis of central sensitization (Klein et al., 2004; Sandkühler et al., 2012) (Table 4). Spinal LTP can be induced in rodents by repeated high‐frequency bursts of electrical stimulation (~100 Hz: Liu & Sandkühler, 1997; Benrath et al., 2005), but also using lower frequencies at 1–10 Hz (Drdla & Sandkühler, 2008; Ikeda et al., 2006; Kim et al., 2015; Terman et al., 2001). Such intense input induces NMDA‐dependent intracellular Ca^2+^ increase in second‐order neurons and astrocytes, with release of brain‐derived neurotrophic factor (BDNF), and activation of purinergic glial receptors (Kim et al., 2015; Retamal et al., 2018; Sandkühler & Gruber‐Schoffnegger, 2012). All these mechanisms can entail signal amplification, but their relation with hyperalgesia remains imperfectly known. Here, we will distinguish studies using low‐frequency stimulation, typically with intradermal electrodes, and studies using high‐frequency stimuli via surface electrodes.

#### 3.4.1 Percutaneous low frequency stimulation (LFS)

Originally described by Koppert et al., (2001), 21 studies used low‐frequency electric stimulation to elicit secondary hyperalgesia. A majority of studies used micro‐dialysis catheters or micro‐neurography needles with 4 mm anode‐cathode distance, stimulating at 0.5–5 Hz. No significant difference was detected when comparing 0.5–1 Hz versus 2Hz versus 5Hz in terms of area, duration or maintenance of 2HA (data from Table 4). LFS intensity is gradually increased during the first 15 min to reach pain intensity reports at 5–6/10, and then kept constant until the end of the experiment. This generally allows maintaining a continuous ongoing pain, upon which secondary hyperalgesia may be assessed. The average hyperalgesic area reported was 39.7 cm^2^ [95% CI: 30–49]. Higher ongoing pain was associated to larger 2HA areas (Table 4), which were in general similar to those under intradermal capsaicin (Geber et al., 2007). The delay to maximal effect was quite variable (range 2–40 min) and 2HA could be maintained for 1.5–5 hr if the stimulation was continuously applied during this period. Both hyperalgesia and allodynia abate in a few minutes after discontinuation of the inducing stimulus (Klede et al., 2003; Koppert et al., 2001). Six of 21 studies reported success rates in inducing 2HA, always at 95%–100%. Excellent test–retest reproducibility of stimulus–response functions (*r* = 0.7) was reported by Geber et al., (2007).

LFS‐induced hyperalgesia was significantly abated by **systemic opioids** including fentanyl, alfentanil, remifentanil and buprenorphine in eight controlled studies (Table 6). Remifentanil lessened 2HA during infusion but generated late hyperalgesia on the post‐infusion period (Lenz et al., 2011; Chu et al., 2012). **Gabapentin** 1200–2600 mg (Boyle et al., 2014; Segerdahl, 2006) and **pregabalin** 300 mg (Chizh et al., 2007) also decreased 2HA/DMA areas and intensities in this model, and one further study reported that 600 mg gabapentin increased the threshold for wind‐up to repetitive electrical stimulation (Enggaard et al., 2010). Systemic **lidocaine** was efficacious in two studies (Koppert et al., 2001; Seifert et al., 2009), as were the **NSAIDs** ketorolac and parecoxib in one single report (Lenz et al., 2001). Modest but significant effects of **propofol**, alone or in combination with remifentanil/ketamine, were also observed (Bandschapp et al., 2010; Nickel et al., 2016). Transcutaneous nerve stimulation (**TENS**‐like) at 20 Hz reduced LFS‐induced hyperalgesia in upper limbs (Nickel et al., 2011), but not at cranial sites (Reindl et al., 2016). LFS‐induced wind‐up was abated by the tricyclic antidepressant **imipramine** in one single study (Enggaard et al., 2001). Neither cholecystokinin (Pahl et al., 2003) nor the anti‐neurokinin‐1 drug aprepitant (Chizh et al., 2007) modified LFS‐hyperalgesia.

The low‐frequency electric model is easy to implement, well‐controlled and provides a large hyperalgesic area with an excellent rate of induction and without serious adverse events. Since this model entails both ongoing pain and 2HA, it may adequately mimic some clinical neuropathic conditions; however, the continuous background pain might hinder the specific assessment of hypersensitivity. Limitations are the slightly invasive nature of electrode placement, discomfort during induction, rapid fall of 2HA/DMA if the electrical stimulus is discontinued (Klede et al., 2003; Koppert et al., 2001), and the need to continuously adjust the stimulation intensity to maintain stable pain ratings (e.g. Boyle et al., 2014).

#### 3.4.2 Cutaneous high‐frequency electrical stimulation (HFS)

High‐frequency stimuli via surface electrodes mimic injury‐induced high‐frequency discharge in altered axons, and are expected to induce central synaptic changes similar to those of real injury (Table 4). The concept was derived from results in animal recordings in nerve and spinal cord (see above), thus enhancing back‐translability. HFS shares many properties with the ID capsaicin model in terms of afferents activated, pain on induction and duration of effect (Henrich et al., 2015). Stimuli are usually delivered through a circular array of 10–16 pin electrodes with array diameter of 6–45 mm (0.3–16 cm^2^ area) designed to activate preferentially superficial nociceptive afferents. Possible influences of application surface and other parameters could not be estimated because of lack of data. Most studies used high‐frequency trains (100 Hz) of 1 s duration, repeated five times at 10‐s intervals, with intensity 10–20 times the electrical detection threshold to single pulses (EDT). One study (Klein et al., 2004) reported larger 2HA areas for 20 versus 10 EDT (56 cm^2^ versus. 38 cm^2^). Xia et al., (2016) reported similar pain amplification induced by 10, 100 and 200 Hz, but higher pain at induction for the 100 Hz model, while Van den Broeke et al., (2019) reported larger hyperalgesic areas to 42 Hz, relative to 100 Hz (80 cm^2^ vs. 50 cm^2^). Hyperalgesia starts rapidly, with maximum effect in 15–60 min, stable for 1–5 hr (Klein et al., 2004, 2006; Pfau et al., 2011; Xia et al., 2016). Two studies using a longer follow‐up reported recovery to baseline in ~24 hr (Klein, et al., 2008; Pfau et al., 2011). When available, responder rates were 80%–100% (Biurrun‐Manresa et al. 2018; ; Pfau et al., 2011). One open label, unblinded study failed to show significant effects of ketamine (0.25 mg/kg) on HFS‐induced 2HA/DMA (Klein et al., 2007).

The main qualities of the HFS model are rapidity and ease of induction and maintenance of 2HA for several hours, the relatively inexpensive equipment and ease of handling. No serious adverse events have been reported. Its main limitation is the unpleasantness of the stimulation (4–8/10) (Magerl et al., 2018; Pfau et al., 2011; Reitz et al., 2016). Because of the relative paucity of information on the behaviour of this model, including to medications, it is likely that our knowledge on its effects will evolve in the next years.

### 3.5 Less prevalent techniques to induce secondary hyperalgesia

Many other pain models have been described that are able to induce secondary hyperalgesia. They are less prevalent than those described above, for reasons including methodological difficulties, inconsistency of results or recent development. Here, we describe models that have provided sufficient data to allow at least a summary of their characteristics and possible practicality.

#### 3.5.1 Procedures activating cold receptors (Supporting Information Table B)

Although cold hypersensitivity is a frequent symptom in patients with neuropathic pain, validated experimental models in humans are scarce. Two main experimental modalities of hyperalgesia induced by cold stimuli have been described, namely the topical application of menthol and the freeze injury model (the latter sharing also properties with burn‐injury and UVB models). Other substances such as mustard's oil and cinnamaldehyde also activate cold‐related receptors such as transient receptor potential ankyrin 1 (TRPA1) (Bandell et al., 2004).

##### The topical menthol hyperalgesia model

Menthol (C10 H20 O) is a cyclic terpene alcohol widely used in anti‐pruritic creams and nasal decongesting formulae, and has been employed as topical application to provoke cold hyperalgesia. Low concentrations (5%–10%) do not produce consistent pain changes (Green, 1992; Yosipovitch et al., 1996), but concentrations of 40% produce pain and local thermo‐mechanical primary hyperalgesia when applied topically (Wasner et al., 2004, Förster et al., 2016). Typical protocols use a 3 × 3 cm soaked gaze with a solution of 30%–40% menthol in 90% ethanol, covered by an adhesive film and applied to the skin during 20 min. Eleven reports using menthol were identified, but only six explicitly tested central sensitization via mechanical 2HA/DMA, and only three provided enough quantitative data. The average area of hyperalgesia was 34.91 cm^2^ [95% CI 18–51] with amplification ratio 3.88 [95% CI 2.04–5.7] (Suppl Table B). Response rates varied from nil to 100%. When 2HA developed, maximum effects were reached immediately and lasted up to 135 min. Sensitization could be prolonged by repeated kindling with cold stimuli (Andersen, Poulsen, et al., 2015).

Menthol‐induced 2HA developed inconsistently: it was systematic in two studies (Andersen, Poulsen, et al., 2015; Binder, et al., 2011); in the others, it developed in a subset of subjects (Namer et al., 2005; Wasner et al., 2004) or could not be measured at all (Hatem et al., 2006; Helfert et al., 2018). Changes in pain thresholds were found reproducible one week apart, but areas of pinprick hyperalgesia were not, and those of DMA could not be determined (Mahn et al., 2014). No controlled studies were identified on the effect of analgesics on menthol‐induced 2HA. Topical menthol was, on the contrary, able to reduce 2HA from cinnamaldehyde (Andersen, et al., 2015).

The main qualities of the menthol model are low discomfort, absence of adverse effects and the fact that cold hypersensitivity may mimic some clinical conditions such as oxaliplatin‐induced neuropathies (Forstenpointner et al. 2018). These advantages may be outweighed by the low success rate and the limited spatio‐temporal amplification.

##### The freeze injury hyperalgesia model

Freezing as a human pain model was first reported one Century ago (Lewis & Love, 1926), but its detailed assessment developed in the 1990s. Beise and colleagues (1998) used a small thermode frozen at −11°C through temperature separation in a Ranque–Hilsch tube. A simpler technique utilizes a copper cylinder of ~2 cm^2^ frozen at −28°C, applied on the anterior part of the forearm for 8–10 s (Chassaing et al., 2006; Kilo et al., 1994; Martin et al., 2019). This procedure was reported to produce a mean 2HA area of 26.85 cm^2^ [95% CI: 13.8–39.9] a surface amplification ratio of 15.23 [95% CI: 7.8–22.7], and a maximum effect 20 hr after application. Secondary hypearalgesia was systematic in three studies; it persisted up to 72 hr in one (Chassaing et al., 2006), and decreased or disappeared after 24h in the other two (Supporting Information Table B). DMA was absent in the two reports that tested it (Kilo et al., 1994; Lötsch & Angst, 2003). Freeze‐induced 2HA was half the size of that obtained with topical capsaicin, in the only study that contrasted both techniques (Kilo et al., 1994).

Three reports examined drug effects on freeze‐induced hyperalgesia. **Dextromethorphan** 30 mg exerted a significant effect on the change of mechanical thresholds within the 2HA region, without modifying the 2HA surface, pain thresholds or pupillary reactions (Martin et al., 2019). Oral **ibuprofen** (400 mg) increased pain thresholds within the 2HA area in one study (Chassaing et al., 2006), while **acetaminophen** (1,000 mg) had no effect on freeze‐induced pinprick hyperalgesia (Chassaing et al., 2006).

The main qualities of the freeze injury are ease of application, low discomfort and long duration of secondary hyperalgesia, making of it a promising technique for drug evaluation needing several testing days. Limitations are the slow development of hyperalgesia, which forces the experimenter to wait one full day between freeze application and testing, as well as the lack of brush‐induced allodynia and the tendency of 2HA to shrink toward the injured area. Serious adverse events have not been described.

##### Activation of TRPA1 receptors: Mustard oil and Cinnamaldehyde

TRPA1 receptors are activated at low temperatures close to cold pain (<17°C) but also by topical application of natural oils such as cinnamaldehyde and mustard oil (Bandell et al., 2004). The specificity of these receptors for cold stimuli remains debated (Weyer‐Menkhoff & Lötsch, 2018, 2019), as they have been also implicated in the perception of heat (e.g. Moparthi et al., 2016).

Mustard oil (Allyl isothiocyanate or AITC) has been extensively used in preclinical studies but only rarely as human model, and only five reports were identified. Topical application for 4–5 min induces strong pain almost immediately, and subsequent 2HA/DMA development was consistently obtained in the few reports available. Koltzenburg et al., (1992), Koltzenburg et al., (1994) induced DMA with mustard oil in 100% of 29 subjects, and could abolish it by blocking large myelinated afferents. 2HA developed inconstantly and was not systematically investigated. Highly variable areas of hypersensitivity across subjects were reported by Sjölund et al., (1999) who also showed reduction of 2HA area (but not DMA) by intravenous adenosine, and a more pronounced, but shorter lasting, 2HA to mustard oil relative to a ‘classical’ thermal‐induced model (47°C, 7 min). Andersen, Elberling, et al., (2017) reported enhanced 2HA/DMA with increasing concentrations from 10% to 50%, without further change at 90%.

Cinnamaldehyde, another powerful activator of TRPA1 receptors, was reported to induce 2HA in two studies (Andersen, et al., 2015; Namer et al., 2005), the latter with average area of pinprick hyperalgesia threefold the application surface (29.43 cm^2^ versus. 9 cm^2^). Co‐application of menthol significantly decreased intensity and 2HA area, which was attributed to a possible combination of segmental spinal inhibition and peripheral receptor‐mediated antagonism between TRPA1 and TRPM8 (Andersen, et al., 2015).

#### 3.5.2 Incisional and pre‐incisional models

Following the description of a plantar incision model in rodents (Brennan et al., 1996), the procedure was translated to humans (Kawamata, Watanabe, et al., 2002). A 4‐mm‐long incision through skin, fascia and muscle in the volar forearm consistently entails 10–12 cm^2^ of pinprick hyperalgesia after 5–15 min, which becomes maximal at 1–2 hr and disappears over the next 6–72 hr (Fißmer et al., 2011; Kawamata, Takahashi, et al., 2002; Kawamata, Watanabe, et al., 2002). The model did not entail brush allodynia. In women, both the intensity and extent of hyperalgesia were found sensitive to the hormonal phases (Pogatzki‐Zahn et al., 2019). Systemic lidocaine previous to incision prevented 2HA, while neither subcutaneous nor systemic lidocaine reverted hyperalgesia once it was fully developed (Kawamata, Takahashi, et al., 2002; Kawamata, Watanabe, et al., 2002).

The incision model appears as a reliable method to induce central sensitization, but its invasive nature represents an obvious disadvantage towards other procedures. A model of non‐injurious sharp mechanical pain using a blade pressing on, but not entering the skin, was proposed to mimic incision‐induced hyperalgesia (Shabes et al., 2016). Although blade and incision‐induced pain descriptors were similar, the size, reproducibility and duration of 2HA to non‐invasive blade were not prominent compared with invasive models.

### 3.6 Models with inconsistent 2HA‐inducing properties

A number of other models with potential to induce 2HA/DMA have received less support from published evidence, or have not been reproduced after their initial description.

#### 3.6.1 Nerve growth factor (NGF) injection

Nerve growth factor (NGF) is a neurotrophin with biological role in the development of small sensory neurons, and participates to the cascade of events leading to lesion‐related neuropathic pain (Khan & Smith, 2015). When injected intradermally, NGF evokes a long‐lasting sensitization of nociceptors with initial heat hypersensitivity, and delayed mechanical hyperalgesia peaking around 3 weeks later (Dyck et al., 1997; Petty et al. 1994; Rukwied et al., ,^2010^, ^2013^). Although 2HA and DMA have been occasionally described (Andresen, et al., 2011), in most studies hypersensitivity was primary, i.e. restricted to the application area (Dyck et al., 1997; Munkholm & Arendt‐Nielsen, 2017; Petty et al., 1994; Rukwied et al., 2010, 2013). Adding NGF to previous UVB irradiation did not influence the hyperalgesic effects (Vecchio, Finocchietti, et al., 2014; Vecchio, Petersen, et al., 2014), while UVB applied three weeks after NGF enhanced the hypersensitivity. NGF has been essentially used to assess muscle hypersensitivity without inducing inflammation.

#### 3.6.2 Injection of hypertonic or acid saline

Injecting hypertonic saline into musculoskeletal structures induces neural firing in A‐delta and C‐nociceptive afferents (Graven‐Nielsen, 2006; Oda et al., 2018). This model is essentially used to mimic muscular or tendon‐related pain, but it has also been shown to induce superficial allodynia to brush or cold stimuli over skin regions surrounding the injection, and might reflect central sensitization (Nagi & Mahns, 2013; Oda et al., 2018; Samour et al., 2017). However, low consistency and concomitant muscle pain greatly decrease the possible impact of this procedure as a convenient 2HA model. A variant of the above considers muscle injection of acidic saline. The acid‐sensing ion channels (ASICs) activate nociceptors in low pH conditions, and acid infusion entails sustained pain behaviour without significant tissue damage (Sluka et al., 2001). Although repeated injections were reported to induce 2HA in animal models (Sluka et al., 2001, 2003) this has not been reproduced with repeated injections in humans (Ernberg et al., 2013; Wang et al., 2017).

#### 3.6.3 Skin irritants, endotoxemia, ciguatoxin

Topical application of **sodium lauryl sulphate**, a skin irritant that releases pro‐inflammatory mediators, was reported to induce both primary and secondary hyperalgesia (Petersen et al., 2010). More robust secondary hyperalgesia to mechanical and cold stimuli was obtained through intradermal injection of **endothelin‐1** (Hans et al., 2007), but neither of the two models has been consistently replicated. **Endotoxemia** via i.v. injection of Escherichia coli lipopolysaccharides induce robust visceral and musculoskeletal hyperalgesia (Benson et al., 2012; Wegner et al., 2014), and may have the potentiality of inducing cutaneous 2HA too (de Goeij et al., 2013), but this has not been specifically explored. **Ciguatoxins** that cause the ‘ciguatera’ condition produce a painful neuropathy characterized by strong cold allodynia (Zimmerman et al., 2013). Experimental injection of ciguatoxin has been used as a surrogate model of cold allodynia in humans (Eisenblätter et al., 2017) but its ability to induce reproducible central sensitization has not been established.

## 4 DISCUSSION

Although more than 15 different human models of secondary hyperalgesia have been described, four classes accounted for more than 90% of published reports. They were based on (1) capsaicin application or injection (reported in >2000 subjects); (2) thermal heat injury (~850 subjects); (3) ultraviolet‐B irradiation (~500 subjects), and (4) repetitive electrical stimuli (~550 subjects). As summarized in Table 5 and Supporting Information **Fig. A**, these models have different profiles in regard of timing of effects, pain intensity during induction, spatiotemporal amplification and proportion of responding subjects. This latter point was reported in less than 50% of studies, giving rise to a high reporting bias.

### 4.1 Differences, strengths and drawbacks of different models

Rather than absolute advantages or disadvantages, each of the principal models appears more or less adapted to different research questions and experimental designs. In what follows, models are discussed in terms of their success rate, timing, spatial and temporal amplification, pain during induction and response to drugs (Table 5).

#### 4.1.1 Success rate

Albeit reported in a minority of studies, success in obtaining hyperalgesia reached 85%–90% for all principal models save the UVB procedure, where half of the accounts either failed to obtain sizeable 2HA areas, or defined them as “barely exceeding the irradiated spot” (Eisenach et al., 2010; Harrison et al., 2004; Morch et al., 2013; Seifert et al., 2008). This model has the particularity of not inducing pain on application. Since nociceptor activity is critical to initiate and maintain central sensitization from peripheral injury (LaMotte et al., 1991; Schmelz et al., 2000, 2009), failure to induce a sustained nociceptive barrage is a likely explanation of the difficulties to obtain 2HA/DMA in the UVB model (Bishop et al., 2009; O’Neill et al., 2015), which remains a robust human model of inflammatory pain, but appears of limited value for the specific study of central sensitization mechanisms (Gustorff et al., 2013).

#### 4.1.2 Spatial and temporal amplification

In order to be useful in pharmacological studies, a model needs (1) to provide an area of 2HA large enough for repeated application of test stimuli, and (2) to induce effects that last long enough to cover peak plasma concentrations. Spatial amplification is obviously highest in models applied to small skin areas (ID‐capsaicin, LFS, HFS). Among the others, amplification ratios were double in topical heat/capsaicin (~18) than thermal injury models (~9), themselves being twice those from UVB models (~4) (Supporting Information Fig. A).

Temporal amplification (ratio between the duration of effect and of conditioning stimulus) is negligible for the “brief thermal” and LFS models, whose effects disappear a few minutes after discontinuation of the inducing stimulus, (Dirks et al., 2003; Hansen et al., 2017; Klede et al., 2003; Koppert et al., 2001). It is also minimal for isolated (non‐kindled) topical capsaicin, but reaches 2–10 fold induction times for ID capsaicin and heat‐injury models (Rasmussen et al., 2015; Simone et al., 1989). Maximal temporal amplification (>10‐fold conditioning times) is attained for intradermal and heat‐kindled capsaicin, UVB, freeze and HFS electric models (Table 5). Temporal amplification, however, cannot be dissociated from the time needed to develop 2HA symptoms, which separates ‘rapid’ from slow‐inducting models. Thus, intradermal capsaicin and HFS provide *both* a sizeable duration of hyperalgesia *and* an immediate onset of effects. Conversely, UVB and freeze‐injury models, although able to generate long hyperalgesic periods, only do so after a latent interval that may last one full day.

A brief duration of hypersensitivity may be especially bothersome when testing DMA, which has intrinsically shorter duration than pinprick hyperalgesia in all models (Geber et al., 2007; Gottrup et al., 2000; LaMotte et al., 1991; Magerl et al. 1998; Pfau et al. 2011; Warnacke et al. 1997). Providing new peripheral input by heating iteratively the skin allows obtaining stable 2HA/DMA during extended periods of time (Dirks et al., 2003; Modir & Wallace, 2010a; Petersen & Rowbotham, 1999). LFS electrical models (0.5–5 Hz) can maintain hyperalgesia for as long as the duration of electrical input; however, the duration of experiments is limited by painfulness of the background stimulus. On the other hand, models allowing a protracted hypersensitivity without pain at induction (UVB, freeze) have the lowest prevalence of 2HA/DMA, most probably because of the limited nociceptive activation they entail (see above).

#### 4.1.3 Pain provoked during induction

Pain provoked during induction may influence subjects’ compliance and attrition rate. Intradermal capsaicin and electrical HFS models induce a very unpleasant stinging sensation, which may reach 7–9/10 on VAS but rapidly abates after stimulus application. On the other hand, the low‐frequency (LFS) electrical models use repetitive noxious stimuli during all the duration of the experiment, and necessarily induce a combination of ongoing pain and secondary hyperalgesia during minutes to hours (Bandschapp et al., 2010; Koppert et al., 2001; Nickel et al., 2011; Wehrfritz et al., 2016). Topical capsaicin and contact‐heat models produce secondary hyperalgesia with only moderate pain on application, which makes them attractive on the condition that the experiments do not exceed several hours. Although no pain at all is induced by freeze and UVB models, this advantage is mitigated by the difficulty to evoke hyperalgesia beyond the territory treated (Harrison et al. 2004; Koppert et al., 1999; O’Neill et al. 2015) and its tendency to shrink towards the irradiated or freezed skin (Chassaing et al., 2006; Eisenach et al., 2010).

### 4.2 Are human models useful surrogates of clinical hyperalgesia?

Human 2HA models can generate an enhanced nociceptive barrage and central sensitization symptoms that are similar to those of neuropathic pain. They cannot mimic the extensive metabolic changes due to neural lesions, nor are they able to model primary central damage responsible for spinal injury or post‐stroke pain. Their translational capacities are indirect, and their value as surrogate models of neuropathic hyperalgesia subject to debate (Van Amerongen et al., 2016; Aykanat et al., 2012; Samuelsson et al. 2011). The quality of models must therefore be validated not only by their capacity to reproduce clinical symptoms, but also by their response to drugs –i.e. they should be responsive to medications active on neuropathic hyperalgesia, and remain insensitive to those without clinical effect (predictive validity). The reverse translation potential of human procedures, i.e. their capacity to inform preclinical models on the adequate endopoints/biomarkers to be used confidently, depends on their ability to respond to pain‐relieving methods that are clinically useful.

#### 4.2.1 Effects of drugs

**FIGURE 4:**
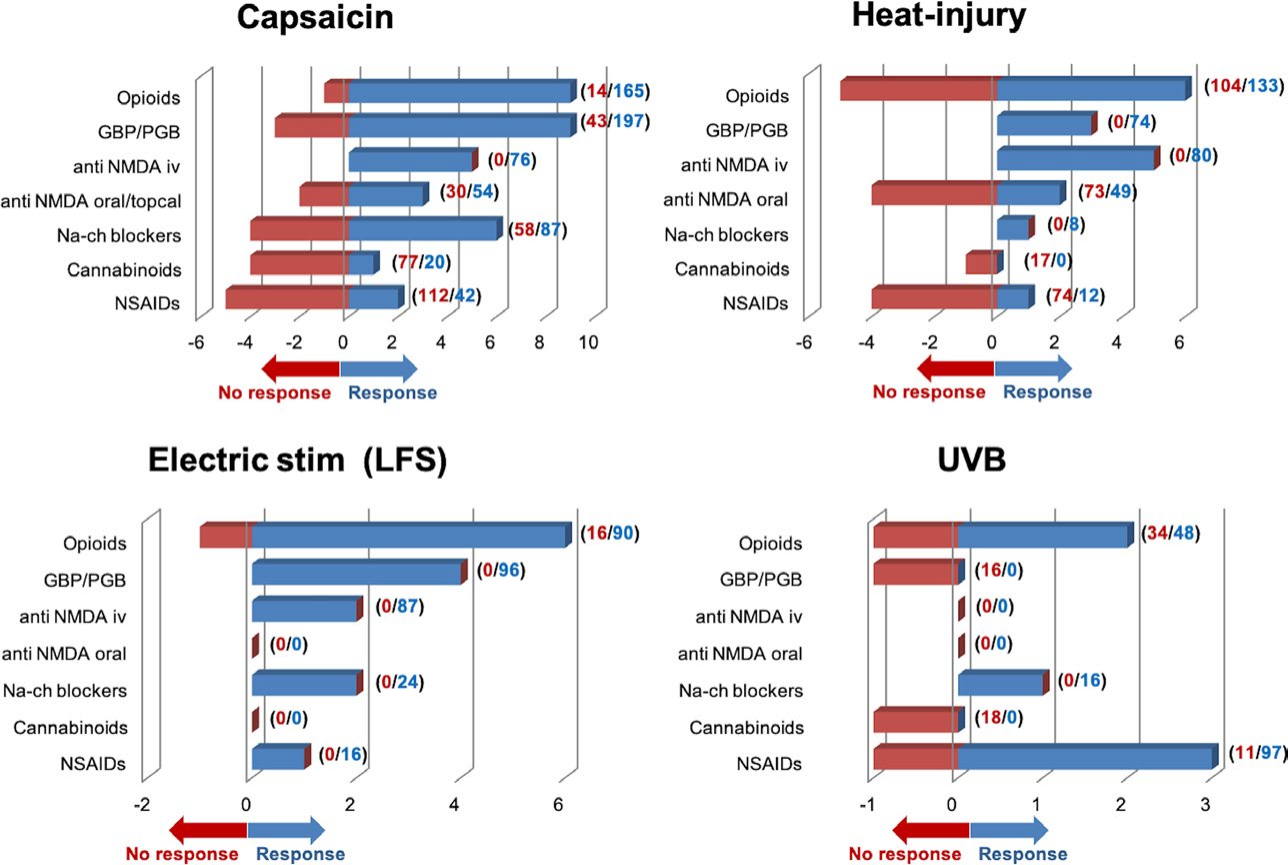
Graphical summary of the models’ responsiveness to major classes of drugs used for neuropathic pain. Studies were classed as “positive” (blue bars) if the drug affected significantly at least one output readout (e.g. 2HA area) versus control/placebo. The number of studies is indicated in abscissae; the number of subjects tested for each drug in negative and positive reports is noted besides each bar. Please note the convergent profile of capsaicin, heat‐injury and electrical models in their response to drugs acting on NP, which contrasts with the UVB profile, mainly reactive to anti‐inflammatory drugs

As summarized in Table 6 and Figure 4, topical and i.d. capsaicin, heat‐injury and LFS electrical models consistently responded to classes of drugs that are clinically valuable for neuropathic pain while remaining largely insensitive to clinically ineffective approaches to central sensitization, including oral NMDA receptor antagonists and NSAIDs. For instance, capsaicin hyperalgesia responded in 73% of reports to drugs clinically accepted for neuropathic pain (gabapentinoids, TCAs, iv ketamine, iv lidocaine) and did not respond in 27% (d = 46; 95% CI 5–85; *p* < 0.05). Although with lower amount of evidence, the capsaicin model also responded to different formulations of clonidine (Eisenach, et al., 2002; Ragavendran et al., 2016), an alpha‐2 agonist used with success in refractory cases of neuropathic pain (Campbell et al., 2012; Schechtmann et al. 2010; Wrzosek et al., 2015).

Drugs may abate secondary hyperalgesia via attenuation of afferent input, central hyperexcitability, or both, and models may help disclosing such mechanisms in humans. Gabapentinoids, intravenous lidocaine and ketamine acted on 2HA but did not affect physiological nociception, as reflected by pain thresholds in normal skin, suggesting a specific reduction of central hyperexcitability. Conversely, opioids and local anaesthetics reduced both secondary hyperalgesia *and* physiological nociception (Andersen et al., 1996; Ilkjaer et al., 1996; Eckhardt et al., 2000, Gottrup et al., 2000; Koppert et al., 2000; Warncke et al., 2000; Dirks et al., 2000; Dirks, Petersen, et al., 2002; Werner et al., 2001; Mathiesen et al., 2006, Wallace, et al., 2002, 2008; Petersen et al., 2014). The facts that opioids may be insufficient to suppress temporal summation, and that they influence hyperalgesia only when the dose permits also a reduction of acute pain, has been put forward to suggest that their action on 2HA is mainly driven by a reduction of afferent nociceptive input, rather than a specific effect on central mechanisms (Brennum et al., 1994; Eisenach et al., 1997; Warncke et al., 1997).

The pattern of response of UVB‐based hyperalgesia differed from that of capsaicin, thermal‐injury and LFS models. UVB 2HA did not respond to gabapentinoids but was sensitive to NSAIDs, which do not show effects on central sensitization in animals except under direct spinal administration (Malmberg & Yaksh, 1992). The surprising sensitivity to NSAIDs of the UVB model may be linked to its inflammatory nature, and the small afferent nociceptive barrage that it triggers. Insufficient ascending input may be inadequate to generate centrally sustained hyperalgesia, and the hyperalgesic state would subside if peripheral input is further reduced due to the peripheral action of NSAIDs such dissimilarities confirm that the UBV procedure is a good model for peripheral inflammation, but not a translational replica of central neuropathic hyperalgesia.

Both capsaicin and heat‐injury models remained insensitive to a number of drugs that worked in animals, but have no or very little efficacy in human neuropathic pain. These include benzodiazepines (Park et al., 1995; Vuilleumier et al., 2013), anti‐histaminics (Wang et al., 2008), lamotrigine (Petersen et al., 2003), T‐type calcium channel blockers (Wallace, et al., 2002), minocycline (Sumracki et al., 2012), melatonin (Andersen, Poulsen, et al., 2015), the anti‐NK1 aprepitant (Chizh et al., 2007), dextromethorphan as add‐on to morphine (Frymoyer et al., 2007), and still others. Also, no anti‐hyperalgesic response was obtained to cannabinoids in the studies reported so far (review De Vita et al., 2018), although one report described a specific decrease in unpleasantness, without changes in pain intensity (Lee et al., 2013).

Some first‐line drugs for human neuropathic pain such as tricyclic antidepressants (TCAs) were only inconsistently active on human 2HA models: TCAs abated wind‐up to repetitive electrical stimuli (Enggaard et al., 2001), but failed to modify 2HA in two capsaicin studies (Eisenach et al. 1997; Wallace, Ridgeway, et al., 2002). The clinical effects of TCAs require a sustained treatment to allow recruitment of downstream mechanisms that cannot be tagged by acute models (Kremer et al., 2016). Adequate testing of the effect of these drugs would need several weeks of continuous treatment, difficult to implement in healthy subjects. These drugs might also target mechanisms that are only activated in chronic conditions and not modelled in volunteers.

Taken together, hypersensitivity from **capsaicin**, **heat‐injury** and **LFS** models responded to drugs clinically accepted for neuropathic pain (gabapentin/pregabalin, iv ketamine, iv lidocaine) in a greater proportion than to drugs with non‐clinical effect in NP, the difference being highly significant (see Table 6).

#### 4.2.2 Methodological issues and controversies

##### Induction of central sensitization

The duration of inducing stimuli, their intensity/dosage and spatial extension, the bending force of testing filaments, the time of testing and various other parameters vary enormously from one study to another, and the same group of investigators can report on the “same” model using different standards. This reflects the lack of consensus on optimal procedures, and together with the variety of output variables hampers generalization of results. Some overall considerations may however be cautiously pondered, for instance that models inducing only mild effects may induce false positive results by responding to drugs with insufficient clinical activity. Relatively ‘soft’ models using low‐concentration topical capsaicin (0.075%–1%) responded to NMDA‐antagonists without proven clinical efficacy such as dextromethorphan or CHF3381 (Duedahl et al., 2005; Mathiesen et al., 2006), while only *intravenous* ketamine was able to counteract ‘strong’ surrogate 2HA models such as rekindled 47°C heat‐injury (compare Ilkjaer et al., 1996, 1997). It appears reasonable that pain models eliciting relatively intense effects may be more appropriate to detect drugs potentially useful in the clinics. It remains to be ascertained whether models eliciting too strong hyperalgesic reactions might also mask the effect of clinically useful agents (Scanlon et al. 2006).

##### Mode of assessment of central sensitization

The ‘optimal’ variable to reproduce clinical data and predict drug effects remains unsettled. Areas of hyperalgesia/allodynia have been the most frequently used readouts, and are often correlated with the evoked pain within the hyperalgesic region. However, these two variables can also be dissociated (Ando et al., 2000; Schifftner et al., 2017; Zheng et al., 2009), and pain within the hyperalgesic region has been reported as more reliable than area size to tag clinically useful analgesia (Ando et al., 2000; Lötsch et al., 2020). Quantifying pain intensity is more subjective and prone to bias than measuring the area of hyperalgesia, which is performed without visual control from subjects (Jensen & Petersen, 2006). On the other hand, the area measured is greatly dependent on the subject's attention and the pressure exerted by the filament (Ringsted et al., 2015), and this may have strong consequences, especially in ‘soft’ models.

A further source of incertitude concerns whether static (pinprick) 2HA or dynamic (brush) allodynia are of equivalent value to predict drug efficacy. These two abnormal percepts result from different central and peripheral mechanisms, may not respond similarly to drugs, and are often dissociated in timing and intensity in both healthy subjects (Cervero et al., 1993; Gottrup et al., 2000; Witting et al., 2000) and neuropathic patients (Gottrup et al., 1998). In all models, pinprick 2HA tends to develop more consistently than DMA, which is often restricted to a smaller area, is less stable, lasts a shorter time and has less distinct borders (Dirks et al., 2003; Geber et al., 2007; Gottrup et al., 2000; LaMotte et al., 1991; Magerl et al., 1998; Pfau et al. 2011; Pöyhiä & Vainio, 2006; Wallace, et al., 2002; Warnacke et al. 1997). Yet, DMA interferes extensively with the patients’ common activities and is considered more troublesome in daily life than pinprick hyperalgesia (Yezierski & Hansson, 2018). Patients with neuropathic pain tend to fear moving, rather than static stimuli (Koltzenburg et al., 1992, 1994; Peyron et al., 1998), and brush‐evoked allodynia correlates with ongoing pain in patients with painful neuropathies (Koltzenburg et al., 1994; Rowbotham & Fields, 1996; Samuelsson et al., 2011), while this has not been shown for pinprick hyperalgesia. Failure to consistently induce DMA may therefore hinder the translational capacities of some experimental models: for instance, of 18 studies reporting significant antihyperalgesic effects of gabapentinoids on surrogate models, 10 failed to induce, report or modify dynamic allodynia.

##### Bias

Many uncontrolled sources of error apply to results of drug trials on hyperalgesia models. At variance with clinical settings, experimental studies often test drug efficacy with a *single* dose administered *before* the inducing stimulus. Blinding may not be feasible if subjects experience subjective effects when administered drugs (ketamine, gabapentinoids, lidocaine), and it would not be reasonable to dismiss a trial as of low quality because of the absence of blinding (Higgins et al., 2011). Dose of inducting agents, skin temperature, pre‐induction pain thresholds and timing of testing are sources of study variability that can hardly be controlled for (Hansen et al., 2017; Liu et al., 1998; Scanlon et al., 2006).

## 5 GENERAL CONCLUSIONS & HINTS FOR FUTURE STUDIES

More than a dozen human surrogate models have been published that mimic aspects of ongoing and evoked neuropathic pain. Despite a significant reporting bias, in particular regarding the percentage of responders and the respective effect on 2HA and DMA, six of these models have been tested in multiple laboratories, and five were found to reliably induce secondary hyperalgesia to pinprick. This may facilitate translation from rodent models (where hypersensitivity to von Frey monofilaments is a frequent readout) to humans using equivalent readouts. More important, crucial benefits should be obtained from *reverse translation*, whereby preclinical models will take advantage of biomarkers that have proven sensitive in human beings. Failure to consistently elicit dynamic allodynia is a yet unsolved drawback, which may hinder the models’ translational capacities. Whether the *areas* of hypersensitivity or the *pain intensity* within these areas should be preferred to model NP symptoms and quantify analgesia remains debatable. For four models, pharmacological profiles have been obtained in sufficient detail to verify similarity to some clinical conditions. Intradermal and high‐dose capsaicin, heat‐injury and LFS models responded in significantly higher proportion to clinically anti‐hyperalgesic drugs than to drugs without proven clinical value, and may be relevant to mimic neuropathic hyperalgesia. The UVB model appears biased towards inflammatory peripheral mechanisms with little contribution of central sensitization. In summary, while it is clearly not possible to model a disease such as neuropathic pain in healthy subjects, there is a sufficient range of validated and easy to use models of key mechanisms and symptoms. Future essays on drug development for neuropathic pain conditions should use them in order to close the translation gap.

Of note, although the initial automatized key‐word based search returned >1,500 papers, this figure became drastically reduced upon multi‐level inspection for relevance. In parallel, automatic search failed to identify a substantial number of reports where terms such as “allodynia”, “hyperalgesia” or “model” were present in the main text but absent from title, abstract or keywords. This may underscore the importance of complementary manual search from bibliographic lists, review papers and grey literature to maximize the number of relevant contributions when dealing with complex topics –and for future researchers looking to update or expand on this review.

## CONFLICT OF INTEREST DISCLOSURE

The authors declare no conflict of interest regarding this work, which received support from the European Union Project EU/EFPIA/Innovative Medicines Initiative Joint Undertaking (IMI‐PAINCARE), grant n° 777,500.

The statements and opinions presented here reflect the author's view and neither IMI nor the European Union, EFPIA, or any Associated Partners are responsible for any use that may be made of the information contained therein. www.imi.europa.eu; www.imi‐paincare.eu. All authors had full access to all of the study data and the corresponding author had the final responsibility for the decision to submit for publication.

## Supporting information

Supplementary Material

Supplementary Material

Supplementary Material
