# Supplementary figures and images for: Human surrogate models of central sensitization: A critical review and practical guide
Source: Eur J Pain. 2021 May 8;25(7):1389–428. doi: 10.1002/ejp.1768 (PMC8360051; doi:10.1002/ejp.1768)

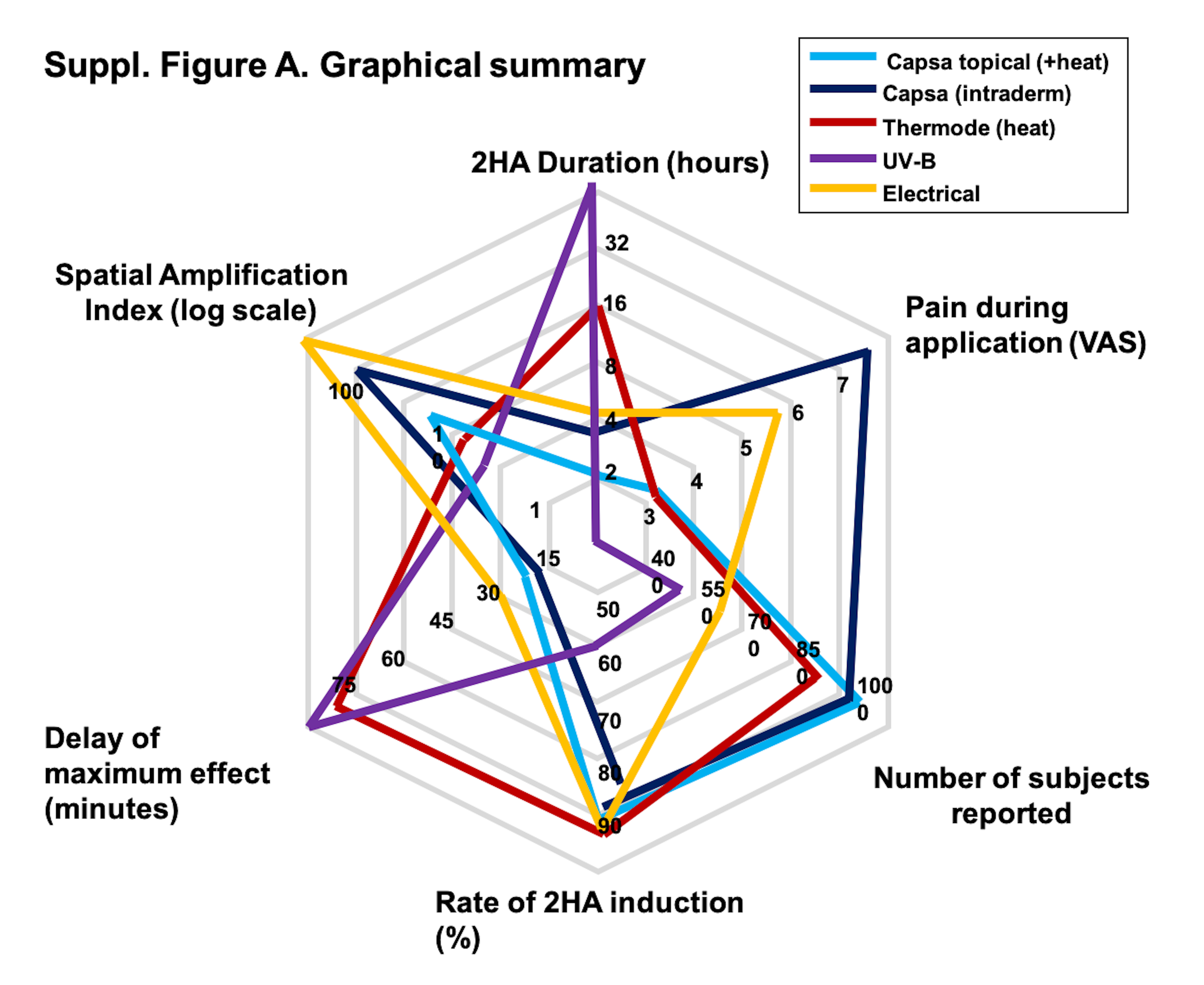

Supplement: Supplementary file 1 — Supplementary Material [file EJP-25-1389-s002.png]
