# Supplementary material for: Human surrogate models of central sensitization: A critical review and practical guide
Source: Eur J Pain. 2021 May 8;25(7):1389–428. doi: 10.1002/ejp.1768 (PMC8360051; doi:10.1002/ejp.1768)
Supplement: Supplementary file 2 — Supplementary Material [file EJP-25-1389-s001.docx]

| **First Author**  **and year** | **N** | **Intensity**  **(in MED)** | **UV-B Radiation Area (cm^2^)** | **2HA area (cm^2^)** | **DMA area (cm^2^)** | **Delay of max effect (hours)** | **Dur. of the 2HA (hours)** | **Responders (%)** | **Spatial Amplification index*** | **Pharma intervention** |
| --- | --- | --- | --- | --- | --- | --- | --- | --- | --- | --- |
| Arendt-Nielsen 2016 | 37 | 3 | 12.5 | 0 | - | - | 3.5 | - | - | - |
| O'neil 2015 | 10 | 3 | 2.56 | 47 | - | - | - | 0 | 18.36 | - |
| Besson 2015 | 25 | 3 | 6.25 | 21.5 | - | 20 | 8 | 92 | 3.38 | Clobazam (+) |
| Vecchio 2015 | 16 | 3 | 12 | 16.8 | - | - | 24 | - | 1.40 | - |
| Vecchio 2014 | 25 | 3 | 12 | 15 | 6 | - | 72 | - | - | - |
| Vecchio 2014 | 24 | 3 | 12 | 47 | - | - | 72 | - | - | - |
| Rossler 2013 | 12 | 3 | 13.8 | 46 | 16 | - | 8 | 100 | 3.33 | Lidocaine (-) |
| Mørch 2013 | 15 | 3 | 1.8 | 0.5 | - | - | 72 | 0 | - | - |
| Weinkauf 2013 | 13 | 3 | 1 | - | - | - | 72 | 0 | - | - |
| Gustorff 2013 | 22 | 3 | 19.6 | 75 | 27 | 20 | 96 | 100 | 3.83 | - |
| Lorenzini 2012 | 13 | 2 | 9 | 21 | - | 20 | 20 | 82 | 2.33 | Paracetamol & Ketorolac (-) |
| Ortner 2012 | 16 | 3 | 13.8 | - | - | - | - | - | - | Paracetamol (+) & Tramadol (+) |
| Rother 2011 | - | - | - | - | - | - | - | - | - | - |
| Lorenzini 2011 | 12 | 2 | 9 | 23 | - | 26 | 48 | - | 2.56 | NSAID (-) |
| Gustorff 2011 | 16 | 3 | 13.8 | 107 | - | 24 | 72 | - | 7.75 | Lidocaine (+) |
| Andresen 2011b | 22 | 3 | 9 | 47 | - | - | 24 | 70 | 5.22 | Buprenorphine (-) & Fentanyl (-) |
| Eisenach 2010 | 14 | 2 | 9 | 20 | 7 | - | - | 100 | 2.22 | Ketorolac (+) |
| Bishop 2009 | 27 | 3 | 1 | 0 | 0 | 24 | - | 0 | - | - |
| Kraft 2008 | 18 | 3 | 0.2 | - | - | - | - | - | - | Cannabinnoid (-) |
| Seifert 2008 | 14 | 3 | 1.76 | - | - | 24 | - | - | - | - |
| Maihofner 2007 | 14 | 3 | 1.76 | - | - | - | - | - | - | Cyclooxygenase inhibit. (+) |
| Sycha 2006 | 6 | 3 | 19.6 | 137.14 | - | - | - | - | 7.00 | Toxbot. A (-) |
| Sycha 2005 | 42 | 3 | 19.6 | 78.74 | - | - | 30 | - | 4.02 | Cox-2 inhibit. (+) |
| Gustorff 2004a | 16 | 3 | 19.6 | 90.85 | - | - | - | 100 | 4.64 | Gabapentine (-) Remifentanil (+) |
| Harrison 2004 | 18 | 3 | 625 | - | - | 48 | - | 0 | - | - |
| Kopper 1999 | 12 | 3 | 19.6 | - | - | - | - | - | - | - |
| Hoffman 1999 | 10 | 3 | 1.76 | - | - | - | - | - | - | - |
| Bickel 1998 | 21 | 3 | 1.76 | - | - | - | - | 0 |  | - |

**Supplementary Table A. Summary of studies using UVB irradiation.** A number of papers also dealt with other models, and are therefore presented in other tables too. Pinprick force is given in grams (1g = 9.8 mN). This model does not entail pain on induction, hence the column “VAS (app)”is absent. MED= Minimum Erythema Dose. *Spatial amplification index = 2HA area/Application area. Note the high variability in 2HA areas and responder rate (0-100%), and the paucity of reports on DMA area.
