# Supplementary material for: Human surrogate models of central sensitization: A critical review and practical guide
Source: Eur J Pain. 2021 May 8;25(7):1389–428. doi: 10.1002/ejp.1768 (PMC8360051; doi:10.1002/ejp.1768)
Supplement: Supplementary file 3 — Supplementary Material [file EJP-25-1389-s003.docx]

| **First Author**  **and year** | **Technique** | **[C]**  **or T°** | **N** | **Dur. of Application** | **Appli. Area (cm^2^)** | **VAS (app)** | **2HA area (cm^2^)** | **DMA area (cm^2^)** | **Delay of max effect** | **Dur. of the 2HA** | **Responders**  **(%)** | **Spatial Amplification Index*** | **Pharma intervention** |
| --- | --- | --- | --- | --- | --- | --- | --- | --- | --- | --- | --- | --- | --- |
| Martin 2019 | Freeze | -28°C | 34 | 8s | 1.76 | - | 39.86 | - | 60 min | 24 h | 50 | 22.65 | Dextrometorphan (+) |
| Chassaing 2006 | Freeze | -28°C | 24 | 8s | 1.76 | - | 17.7 | - | 20 h | 72 h | 100 | 10.06 | Acetaminophen (-) & Ibuprofen (+) |
| Kilo 1994 | Freeze | -28°C | 9 | 8s | 1.76 | - | 23 | 1.2 | 22 h | - | - | 13.07 | - |
| Helfert 2018 | Menthol | 40% | 16 | 20 min | 9 | - | - | - | - | - | - | - | - |
| Andersen 2015 | Menthol | 40% | 20 | 20 min | 9 | 6.7 | 22 | 8 | 0 | 75 min | 75 | 2.44 | - |
| Mahn 2014 | Menthol | 40% | 10 | 20 min | 9 | 7.9 | 32 | -** | - | - | 100 | 3.56 | - |
| Binder 2011a | Menthol | 40% | 12 | 20 min | 9 | 2.1 | 50.74 | - | 135 min | 225 min | 100 | 5.64 | - |
| Namer 2005 | Menthol | 40% | 10 | 20 min | 12.5 | 3.1 | - | - | - | - | 40 | - | - |
| Wasner 2004 | Menthol | 40% | 10 | 20 min | 12.5 | 3 | - | - | - | - | 20 | - | - |

**Supplementary Table B.** **Summary of studies on cold-based models**. Some of the studies in this table also dealt with other models and may therefore be presented in other tables too. VAS (app)= pain during application on a 0-10 scale. *Spatial amplification index= 2HA area/Application area

**= DMA obtained in 40% of subjects, who were unable to delineate a clear area.
